# White Matter Microstructure and Its Relation to Longitudinal Measures of Depressive Symptoms in Mid- and Late Life

**DOI:** 10.1016/j.biopsych.2019.06.011

**Published:** 2019-11-15

**Authors:** Xueyi Shen, Mark J. Adams, Tuula E. Ritakari, Simon R. Cox, Andrew M. McIntosh, Heather C. Whalley

**Affiliations:** a Division of Psychiatry, University of Edinburgh, Edinburgh, United Kingdom; b Centre for Cognitive Ageing and Cognitive Epidemiology, University of Edinburgh, Edinburgh, United Kingdom; c Department of Psychology, University of Edinburgh, Edinburgh, United Kingdom

**Keywords:** Big data, Depression, Longitudinal, Neuroimaging, UK Biobank, White matter microstructure

## Abstract

**Background:**

Studies of white matter microstructure in depression typically show alterations in individuals with depression, but they are frequently limited by small sample sizes and the absence of longitudinal measures of depressive symptoms. Depressive symptoms are dynamic, however, and understanding the neurobiology of different trajectories could have important clinical implications.

**Methods:**

We examined associations between current and longitudinal measures of depressive symptoms and white matter microstructure (fractional anisotropy and mean diffusivity [MD]) in the UK Biobank Imaging Study. Depressive symptoms were assessed on two to four occasions over 5.9 to 10.7 years (*n* = 18,959 individuals on at least two occasions, *n* = 4444 on four occasions), from which we derived four measures of depressive symptomatology: cross-sectional measure at the time of scan and three longitudinal measures, namely trajectory and mean and intrasubject variance over time.

**Results:**

Decreased white matter microstructure in the anterior thalamic radiation demonstrated significant associations across all four measures of depressive symptoms (MD: βs = .020–.029, *p*_corr_ < .030). The greatest effect sizes were seen between white matter microstructure and longitudinal progression (MD: βs = .030–.040, *p*_corr_ < .049). Cross-sectional symptom severity was particularly associated with decreased white matter integrity in association fibers and thalamic radiations (MD: βs = .015–.039, *p*_corr_ < .041). Greater mean and within-subject variance were mainly associated with decreased white matter microstructure within projection fibers (MD: βs = .019–.029, *p*_corr_ < .044).

**Conclusions:**

These findings indicate shared and differential neurobiological associations with severity, course, and intrasubject variability of depressive symptoms. This enriches our understanding of the neurobiology underlying dynamic features of the disorder.

SEE COMMENTARY ON PAGE 734

Major depressive disorder (MDD) is a disabling disorder with a heritability of approximately 37% 1, 2 and 16% lifetime risk (3). It is a heterogeneous illness 4, 5, often studied in modest sample sizes (6). These limitations have led to inconsistent findings (7) and to an uncertain relationship between quantitative measures of depressive symptoms and associated neurobiology.

A possible contributor to heterogeneous imaging findings in MDD is the longitudinal variability of depressive symptoms (8). This dynamic property is rarely captured by most imaging investigations, but it potentially has important implications in terms of both understanding disease heterogeneity and having clinical relevance 9, 10, 11. Comparing the neurobiological associations of current and longitudinal depressive symptoms is important for identifying causal mechanisms underlying depressive symptoms as well as identifying predictors of symptom onset, variability, and progression over time. Brain structural measures have previously been found to be associated with stable depressive conditions over time such as self-declared lifetime depression 12, 13, 14. Studies on symptomatic changes, however, have also suggested that brain structural measures, such as cortical thickness and volume, can vary along with the fluctuations of current symptoms (15).

However, identifying the neural associations of dynamic longitudinal features of depressive symptoms within a single well-powered imaging study has to date been challenging owing to a lack of suitable data. To identify imaging correlates of depressive symptoms, a large imaging sample with repeated and consistently assessed measures of depressive symptoms is required. Such studies are resource intensive, take many years to complete, and rarely provide the data required in sufficient numbers of participants. The UK Biobank Imaging Study (https://imaging.ukbiobank.ac.uk/), however, is a rare exception and is by far the largest neuroimaging cohort with longitudinal depressive symptom data to date.

In the UK Biobank Imaging Study, depressive symptoms were assessed on up to 4 separate occasions across a time span of 5.89 to 10.69 years. One depressive symptom assessment was conducted at the same occasion as the magnetic resonance imaging evaluation. Based on all available measurements, we generated 4 measures of depressive symptoms under 2 categories. The first category contained a cross-sectional assessment of depressive symptom severity at the same time as the imaging assessment, representing the current levels of symptoms of depression. The second category contains 3 measures derived from multiple assessments. These longitudinal measures are the longitudinal slope of depressive symptoms within an individual over time up until the imaging assessment (this was used as a proxy for assessing the longitudinal course of depressive symptoms over time), the mean level of depressive symptoms averaged over all measures, and the standard deviation of depressive symptoms as a measure of within-participant variability over time.

In the current study, we first investigated the associations between these measures of depressive symptoms and white matter microstructure because of the growing evidence of an association between mood disorders and reductions of white matter microstructure in the limbic system, especially in the thalamic radiations 16, 17. These networks contain important tracts involved in emotional processing (18) and regulation (19). Lower microstructural integrity in these regions are typically associated with the onset and severity of MDD (20). In the current study, 19,345 people with diffusion tensor imaging (DTI) data were included to test the association between white matter microstructure and the cross-sectional and longitudinal measures of depressive symptoms (21). Second, to explore potential differential contributions, we used stepwise regression models to test which brain regions in particular were associated with the cross-sectional measure of current symptoms, over and above those associated with longitudinal measures, and conversely which ones were particularly sensitive to longitudinal measures, over and above those associated with current symptoms from the cross-sectional measures. Finally, because these dynamic features may reflect differential clinical and behavioral profiles, we also tested correlations between the above cross-sectional and longitudinal measures themselves and their associations with 12 potentially MDD-relevant behavioral, demographic, and cognitive measures.

## Methods and Materials

### Participants

UK Biobank initially recruited 500,000 people across the United Kingdom (22) and has had an ongoing program of repeated behavioral assessments that were first conducted at baseline. In addition, UK Biobank has begun a brain imaging study of 100,000 individuals (21). For the current analyses, the most recent release of imaging data was used (October 2018). This sample included data from 19,345 individuals after preprocessing and initial quality control conducted by the UK Biobank team (23). The mean age of participants was 63.06 years (SD = 7.44), and 47.14% were men. We then conducted further data quality control of the remaining participants by removing outliers 16, 17. Finally, the imaging data were merged with other relevant behavioral variables (Supplemental Figure S1).

UK Biobank data acquisition was approved by the research ethics committee (reference 11/NW/0382). The analysis and data acquisition for the current study were conducted under application 4844. Written consent was obtained for all participants.

### Depressive Symptoms

Depressive symptoms were measured by a 4-item Patient Health Questionnaire-4 (PHQ-4) (24). The PHQ-4 has an area under the curve of 0.79 for its correlation with depression diagnosis (25). In other work, this measure was significantly associated with measures of disability (26) as well as risk factors for depression (24,27) (see http://biobank.ctsu.ox.ac.uk/crystal/label.cgi?id=100060 and Supplemental Methods).

The PHQ-4 was assessed repeatedly up to 4 times. Time points included 1) the first assessment visit (2006–2010, *n* = 19,231), 2) a repeat visit on a subsample (2012–2013, *n* = 4535), 3) the imaging visit (2014–2017, *n* = 19,113), and finally 4) the online follow-up (2015–2018, *n* = 14,155) (http://biobank.ctsu.ox.ac.uk/crystal/label.cgi?id=100060).

**Figure 1:**
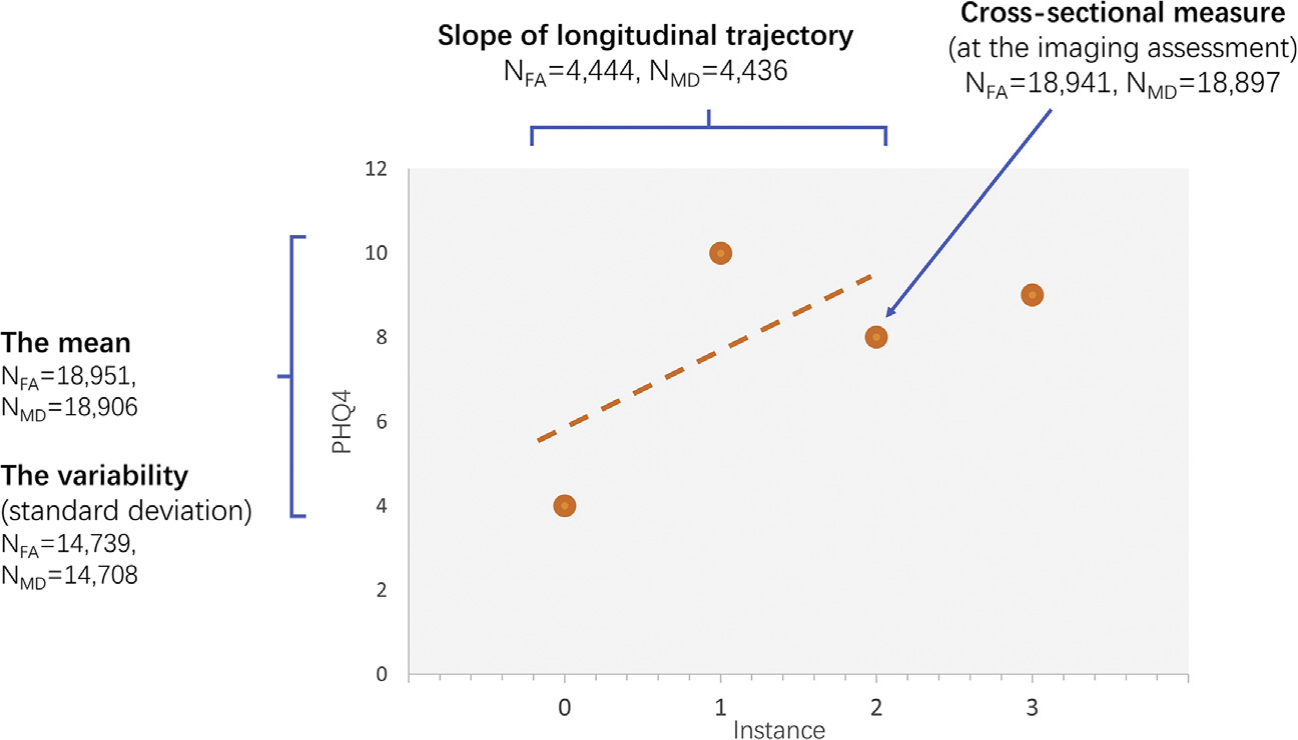
The measures for depressive symptoms generated for this study. The x-axis represents each instance (0 = baseline, 1 = first repeated assessment, 2 = imaging assessment, and 3 = online follow-up, consistent with the coding for instances in UK Biobank [http://biobank.ctsu.ox.ac.uk/showcase/instance.cgi?id=2]). There were 4 measures generated: 1) cross-sectional measurement for depressive symptoms acquired with imaging assessment, 2) linear growth curve denoting longitudinal trajectory of depressive symptoms derived from 3 time points up until the imaging assessment, 3) mean of depressive symptoms generated based on at least 2 multiple assessments, and 4) variability of depressive symptoms, which was the SD of at least 3 time points. FA, fractional anisotropy; MD, mean diffusivity; PHQ4, Patient Health Questionnaire-4.

**Figure 2:**
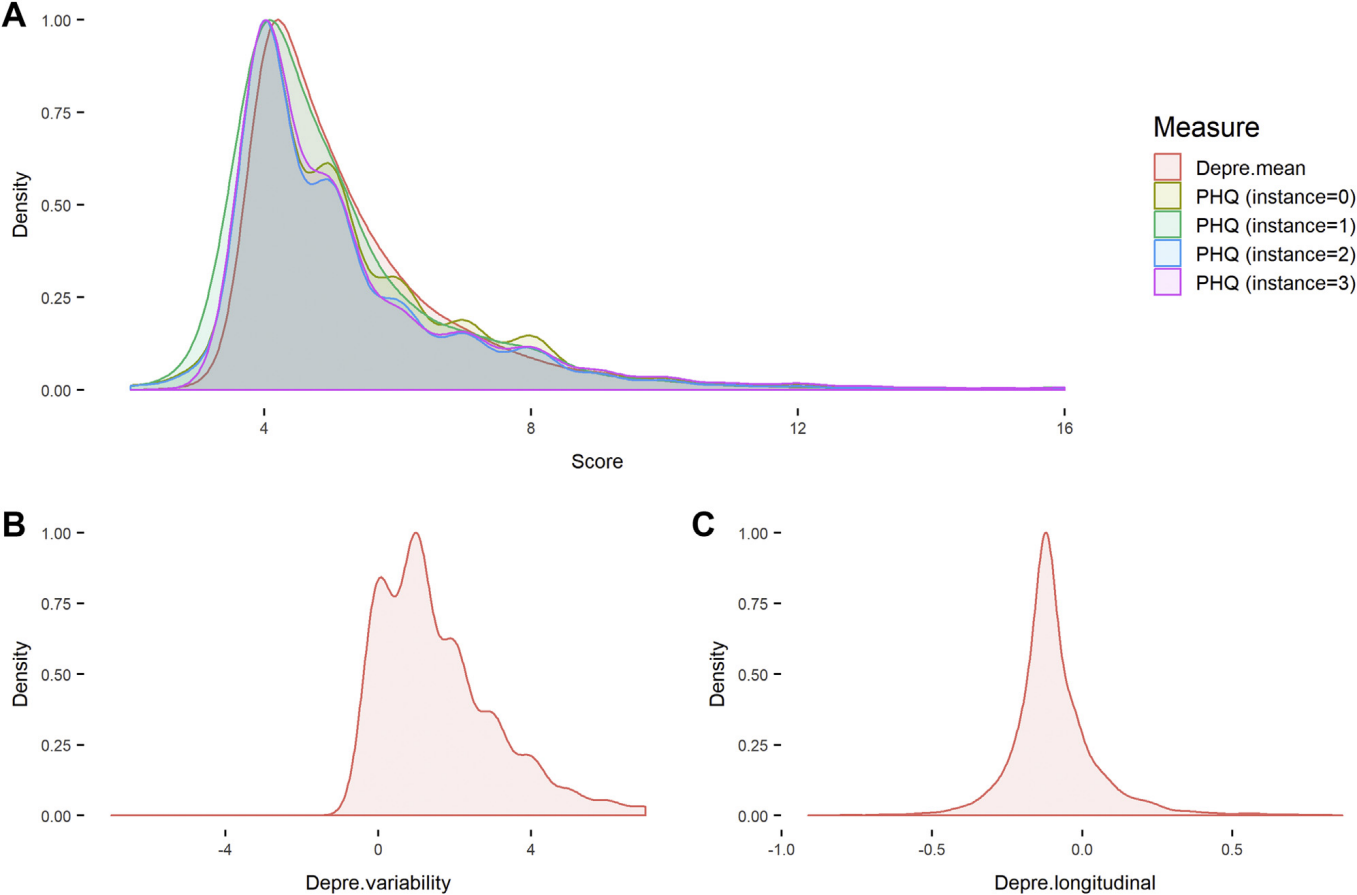
Distributions of Patient Health Questionnaire-4 (PHQ) and derived measures for depressive symptoms. **(A)** Density plot for all 4 time points of assessment for PHQ. Instance = 2 was for imaging assessment, and the depressive level acquired from this instance was used as a baseline one-time measure for depressive symptoms. The mean depressive level over a minimum of 2 time points of assessment (Depre.mean) is also presented in this panel. **(B)** Density plot for instability of depressive symptoms (Depre.variability). **(C)** Density plot for the distribution of slope for the longitudinal trajectory of depressive level over 4 instances (Depre.longitudinal). The density plots were made using the geom_density function in ggplot2 with a smoothness adjustment of 2.

Based on the repeated PHQ-4 measures, we generated 4 measures of depressive symptoms (Figure 1, Supplemental Figure S1, and Supplemental Table S1), with *n* included in the analyses reported in parentheses (for fractional anisotropy [FA] and mean diffusivity [MD]). One measure was a single PHQ-4 score measure acquired at the time of imaging assessment, representing the current depressive symptoms (*n*_FA_ = 18,941, *n*_MD_ = 18,897). Three other measures were generated based on multiple assessments, including 1) the estimated longitudinal slope of depressive symptoms from the initial recruitment up until the imaging assessment, using a linear growth curve model, with positive values indicating a relative increased (i.e., worsening) progression over time and negative values indicating a relative decrease of depressive symptoms (i.e., improvement) over time (*n*_FA_ = 4444, *n*_MD_ = 4436); 2) the mean (*n*_FA_ = 18,951, *n*_MD_ = 18,906); and 3) variability of depressive symptoms across all available assessments (*n*_FA_ = 14,739, *n*_MD_ = 14,708), where mean depression level was the PHQ-4 average over 2 or more time points and variability of depressive symptoms was the standard deviation of PHQ-4 scores over a minimum of 3 time points. Details of the growth curve model estimation are provided in the Supplemental Methods and Figure 2.

### Imaging Data

We used the quality-controlled imaging-derived phenotypes from the DTI assessments released by the UK Biobank Imaging Study. Details of the dataset can be found in the protocol documentation (https://biobank.ctsu.ox.ac.uk/crystal/docs/brain_mri.pdf) and in 2 published protocol articles 21, 23. Major processing steps are described here in brief.

Two DTI microstructure measures were estimated after preprocessing 28, 29. FA and MD were generated using DTIFIT (30). A probabilistic tractography-based method using the AutoPtx package from FSL was used to map 27 major tracts over the whole brain (21). The processed tracts included 12 bilateral and 3 unilateral tracts (Supplemental Figure S2 and Supplemental Methods). Masks of tracts derived from FA data were used to locate the tracts on MD. Weighted means of DTI measures for each tract were generated.

Three newly developed neurite orientation dispersion and density imaging measures were also generated using the AMICO tool as supplementary measures to the classic DTI measures (31). These measures depict additional sources of variation such as neurite density, extracellular water proportion, and morphology of tract organization. For brevity these findings are not presented in the current article, but for completeness they are detailed in Supplemental Figure S3.

### MDD-Related Behavioral Data

We also examined the association of each depressive symptom measure with an additional 12 potentially relevant behavioral, demographic, and cognitive measures that have been found to be associated with MDD or depressive symptoms in previous studies 32, 33, 34.

The 12 measures included neuroticism, self-declared age of onset for depression, sociodemographic variables (household income, educational attainment, and Townsend index), lifestyle measures (insomnia and smoking status), and physical measures (body mass index and hand grip strength). Finally, we also examined the association of each symptomatic variable with measures of recent pain and general cognitive functions (a general *g* factor generated by the first unrotated principal component of several cognitive tasks). More details are provided in Supplemental Methods.

### Statistical Methods

#### Associations Between Measures of Depressive Symptoms and White Matter Microstructure

Before analysis was performed, outliers were removed (16). This was achieved by performing a separate principal component analysis for each DTI measure on the overall sample of 19,345; those whose scores for the first principal component were outside of ±3 SDs from mean were removed (16). This resulted in a maximum of 19,262 participants remaining (see Supplemental Figure S1) for further analysis.

We tested associations between each measure of depressive symptoms and the white matter microstructure measures with increasing level of regional detail: first using whole brain metrics, then using 3 tract subsets (using the categories association/commissural fibers, thalamic radiations, and projection fibers) (see Supplemental Figure S1) (16), and finally examining individual tracts separately. Indices for the whole brain and tract subsets were derived as before from the score of the first unrotated principal component for each microstructural metric (16). These are denoted as general variance over all tracts (gTotal), general variance for tract subset of association/commissural fibers (gAF), general variance for thalamic radiation (gTR), and general variance for projection fibers (gPF) (Supplemental Figure S4 and Supplemental Table S2).

The glm function in R was used to test for associations between each symptomatic measure and each unilateral tract. We used the R package lme for the analysis of bilateral tracts [where hemisphere was controlled and each tract was modeled as a repeated measure (35)]. Age, age squared, sex, head position in the scanner (on x-, y-, and z-axes), and magnetic resonance imaging site were set as covariates. Other covariates included smoking status and alcohol consumption before the time of imaging assessment to control for depression-related behavioral patterns that may influence brain structure. We also adjusted for recent stressful life events within 2 years before the imaging assessment in order to have more accurate estimations of the associations related to inherent mood variability. Each of the covariates is described in the Supplement. For completeness, we also report results that did not control for smoking status, alcohol consumption, or stressful life events in Supplemental Figure S5.

#### Identifying the Separate White Matter Associations of Trait and State Measures of Depressive Symptoms

Cross-sectional measures of symptom severity, as an index of current state, and the longitudinal mean and variability, as indices of the trait, may be expected to be correlated but also potentially distinctive. Therefore, we investigated which regions were more specifically associated with current state by evaluating white matter associations with depressive symptom severity measured at the time of the imaging assessment while adjusting for the longitudinal mean and variability symptom estimates. We also explored the reverse, testing which regions were associated with longitudinal trait features (mean and variability) over and above the single cross-sectional measure of current symptoms obtained at the time of the imaging assessment.

We utilized a stepwise regression method, using the analysis of variance function in R. This tested whether the added independent variables significantly contributed to a reduction in the residual term of the model. First, we undertook a comparison of models to determine the unique contribution of cross-sectional symptom severity associations with imaging measures. Here we define the null hypothesis (H0) and alternative hypotheses (H1) models:

H0model:imagingvariables∼covariates+mean+variability

H1model:imagingvariables∼covariates+mean+variability+cross-sectional

By comparing the H0 and H1 models, a significant reduction of residual in the H1 model compared with the H0 model would indicate an identifiable contribution of the cross-sectional symptoms measure to the model and, therefore, a state-specific association between depressive symptoms and white matter microstructure.

Similarly, to test which imaging variables the longitudinal measures contribute significantly over and above the cross-sectional measures, the H0 and H1 models were defined as follows:

H0model:imagingvariables∼covariates+cross-sectional

H1model:imagingvariables∼covariates+cross-sectional+mean+variability

#### Associations Between Measures of Depressive Symptoms and Behavioral Variables

We first analyzed the correlations within the 4 measures of depressive symptoms themselves, and then conducted analysis on the associations between these measures and other behavioral variables. For the associations between measures of depressive symptoms and behavioral variables, they were tested while controlling for age, age squared, sex, and magnetic resonance imaging site using the glm function in R (version 3.2.3). The *p* values were false discovery rate corrected using the p.adjust function (*q* value < .05) applied for 4 (symptom measures) × 12 (behavioral variables) = 48 tests. All effect sizes throughout are standardized. For the logistic regressions, the effect sizes are reported as log-transformed odds ratios.

## Results

### Associations Between Measures of Depressive Symptoms and White Matter Microstructure

**Figure 3:**
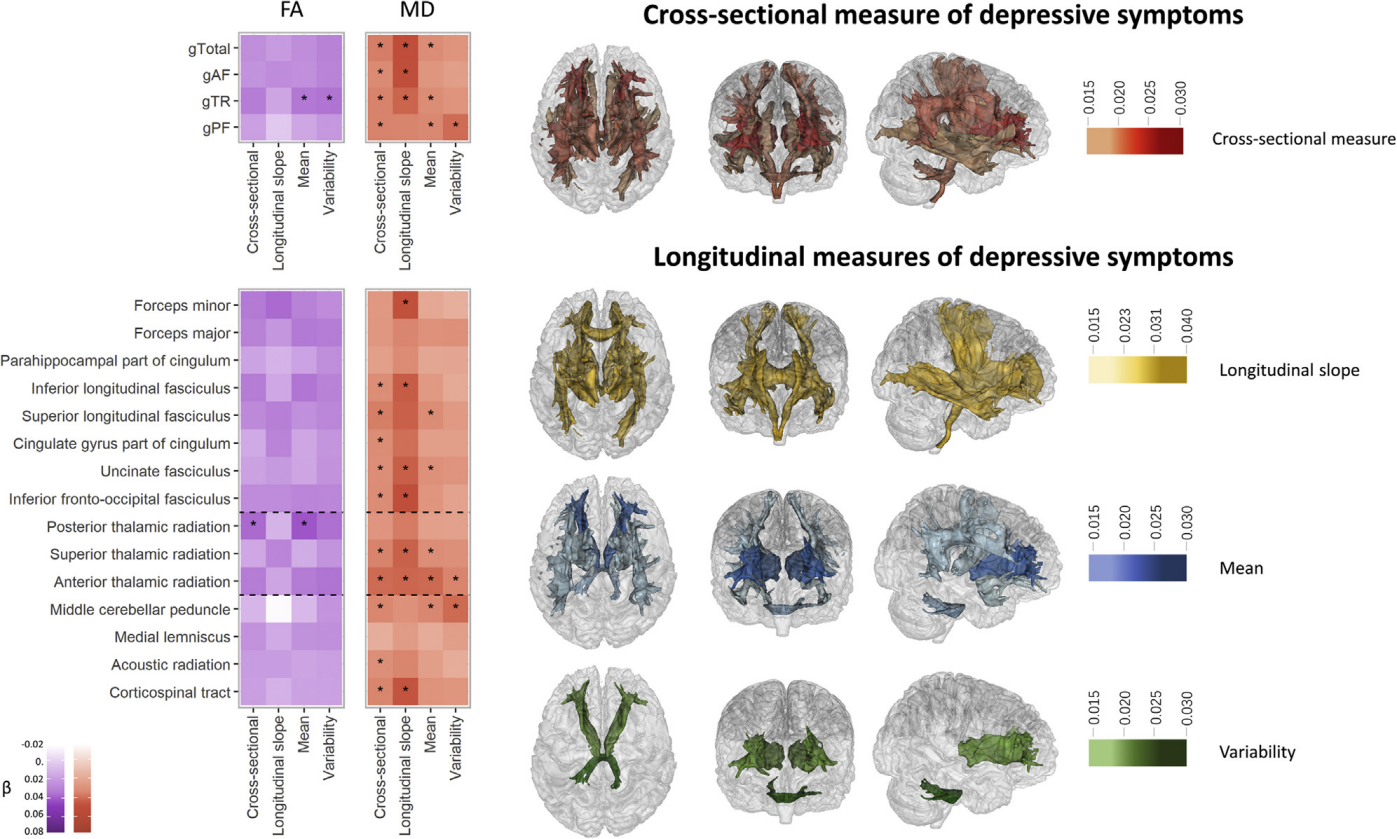
Associations between cross-sectional depressive symptoms and diffusion magnetic resonance imaging (heatmap) and the map for significant regions (brain map). For the heatmap, color depth represents the standard effect size of a measure. Because fractional anisotropy (FA) has a negative direction with mean diffusivity (MD) here in this figure, the effect sizes for FA are reversed (× −1). The results are separated into two sections. The upper sections show the results for g measures, and the lower sections show the results for individual tracts. To aid comprehension, in the lower part where results of tracts are shown, checks are divided into 3 categories by dashed lines because the tracts are in different subsets (i.e., association fibers, thalamic radiations, and projection fibers (see Methods and Materials). Significant associations after false discovery rate correction (*p*_corr_ < .05) are marked with an asterisk. For the brain map, significant tracts are shown in red for the ones associated with cross-sectional measure, yellow for the ones associated with longitudinal slope, blue if associated with the mean, and light green if associated with variability. gTotal, general variance over all tracts; gAF, general variance for tract subset of association/commissural fibers; gTR, general variance for thalamic radiation; gPF, general variance for projection fibers.

The anterior thalamic radiation was the only structure that was significantly associated with all 4 measures of depressive symptoms (βs = .028–.030, *p*_corr_ < .049) (see Figure 3 and Supplemental Table S3). The largest effect sizes for the white matter associations, however, were shown with measures longitudinal trajectory (βs = .030–.040 for significant associations, *p*_corr_ < .049). Cross-sectional, mean, and variability of depressive symptoms generally had lower effect sizes than the longitudinal slope associations (βs = .015–.029 for significant associations, *p*_corr_ < .044). The main results for each of the 4 measures of depressive symptoms are described in detail below. Results without controlling for smoking status, alcohol consumption, and stressful life events demonstrated similar patterns of results to the main findings controlling for these factors (see Supplement).

### Cross-sectional Measure of Depressive Symptoms

Higher general MD in all tract categories, including gTotal, gAF, gTR, and gPF, were positively associated with cross-sectional depressive symptoms (βs = .019–.024, *p*s_corr_ = .009–.002).

MD in a total of 10 individual tracts was found to be associated with cross-sectional measures of depressive symptoms. Higher cross-sectional symptom severity was positively associated with higher MD in the acoustic radiation, anterior thalamic radiation, inferior longitudinal fasciculus, inferior fronto-occipital fasciculus, uncinate fasciculus, superior thalamic radiation, corticospinal tract, superior longitudinal fasciculus, cingulate gyrus part of cingulum, and middle cerebellar peduncle (βs = .015–.028, *p*s_corr_ = .041–1.47 × 10^−4^).

For FA, no significant associations were found for the global measures (whole brain and tract categories) to be associated with the cross-sectional symptom severity (*p*_corr_ > .051). For the individual tracts, only FA in posterior thalamic radiation was associated with higher cross-sectional measure (β = −.024, *p*_corr_ = .011).

### Longitudinal Measures of Depressive Symptoms: Longitudinal Slope, Mean, and Variability

Greater longitudinal worsening of symptoms was positively associated with gTotal, gAF, and gTR for MD (βs = .031–.040, *p*s_corr_ = .025–.009).

Both higher mean and greater variability of depressive symptoms were found to be positively associated with higher MD in gPF (mean: β = .020, *p*_corr_ = .009; variability: β = .029, *p*_corr_ = .002). Additional associations with the mean level of depressive symptoms were seen in global MD (β = .018, *p*_corr_ = .013) and MD in the subset of gTR (β = .018, *p*_corr_ = .009).

Tract-specific analysis showed that greater longitudinal increase of depressive symptoms was positively associated with higher MD in the anterior thalamic radiation, corticospinal tract, inferior fronto-occipital fasciculus, inferior longitudinal fasciculus, superior thalamic radiation, uncinate fasciculus, and forceps minor (βs = .030–.038, *p*s_corr_ = .049–.030).

For mean and variability measures, MD in individual tracts that was positively associated with both higher mean and variability of depressive symptoms was seen in the anterior thalamic radiation (mean: β = .029, *p*_corr_ = 1.47 × 10^−4^; variability: β = .020, *p*_corr_ = .030) and middle cerebellar peduncle (mean: β = .019, *p*_corr_ = .038; variability: β = .029, *p*_corr_ = .008). Additional associations that were found for the mean of depressive symptoms were shown for higher MD in superior longitudinal fasciculus, uncinate fasciculus, and superior thalamic radiation (βs = .015–.017, *p*s_corr_ = .044–.038).

FA in gTR was negatively associated with mean level and within-participant variability of depressive symptoms over time (βs = −.021 and −.022, *p*s_corr_ = .045 and .045, respectively). For individual tracts, the only association was found between FA in posterior thalamic radiation and mean depressive symptoms (β = −.029, *p*_corr_ = 7.85 × 10^−4^). No significant association between tract-specific FA variation and longitudinal slope or variability of depressive symptoms was found (*p*_corr_ > .055).

### Assessing the Associations of Current Symptoms After Adjustment for Longitudinal Measures and Vice Versa

**Figure 4:**
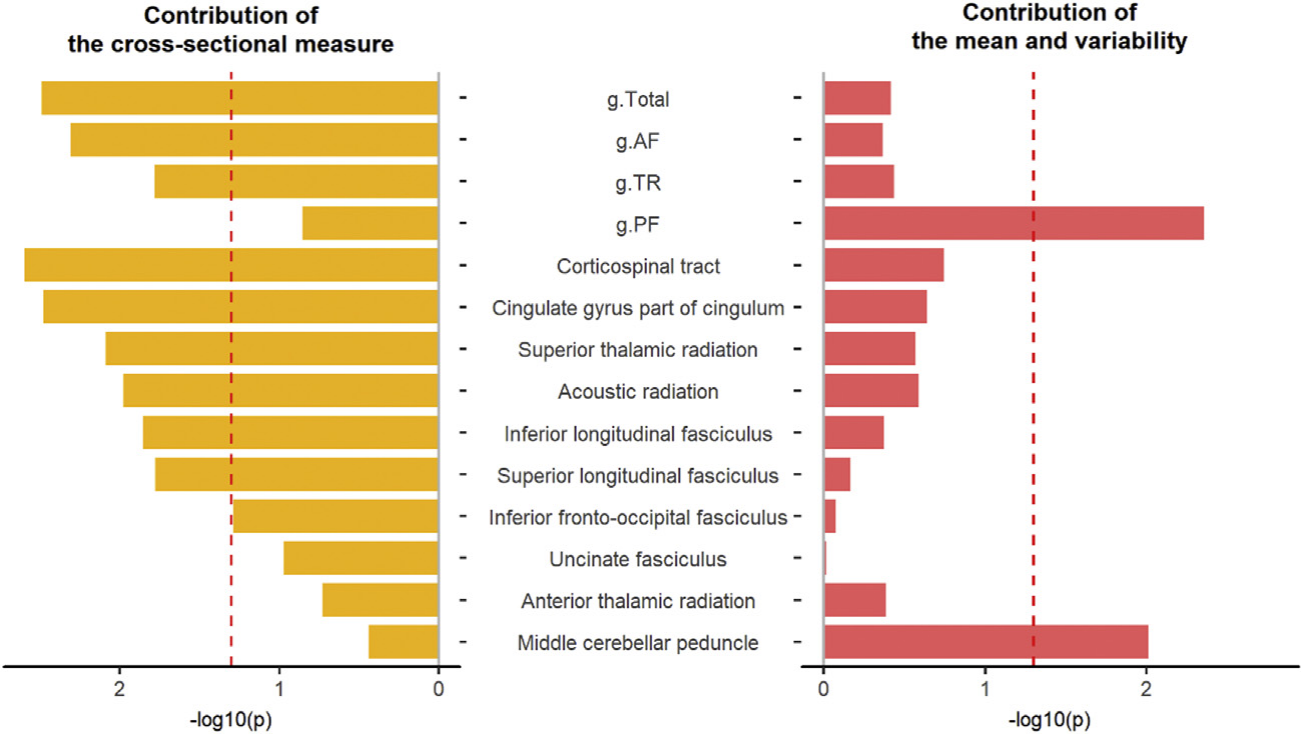
Contributions by the cross-sectional measure and by the mean and variability of depressive symptoms on mean diffusivity. The y-axis represents the variables that were found associated with cross-sectional depressive symptoms. The x-axis represents the −log10(*p*) for whether adding a relevant measure(s) of depressive symptoms adds significantly more variance explained. For the left panel, a null hypotheses model with independent variables that include the mean, variability, and cross-sectional measure was compared with an alternative hypothesis model with only the mean and variability as the dependent variables. Bars to the left of the red dashed line (the *p* = .05 line) indicate a significant contribution of the cross-sectional measure. The opposite is true for the right panel, in which bars to the right of the red dashed line indicate a significant contribution of the mean and variability for mean diffusivity in these neuroimaging variables. The following measures of white matter microstructure are used: g.AF, general variance for tract subset of association/commissural fibers; g.PF, general variance for projection fibers; g.Total, global variance of mean diffusivity; g.TR, general variance for thalamic radiations.

As shown in Figure 4 (also in Supplemental Tables S4 and S5), a significant additional contribution of the cross-sectional symptom measure, over and above the longitudinal measures, was found for MD in gTotal, gAF, and gTR (*Fs*_1,14686_ for H0–H1 model comparison = 5.75–8.66, *p*s = .017–.003). Additional contribution by the cross-sectional symptom measure was also shown for individual tracts including the superior longitudinal fasciculus, superior thalamic radiation, inferior longitudinal fasciculus, corticospinal tract, acoustic radiation, and cingulate gyrus part of cingulum (χ^2^_1_ values = 5.73–9.12, *p*s = .017–.003).

Conversely, MD in gPF showed a significant additional contribution from the mean and variability measures over the cross-sectional measure (*F*_2,14686_ = 5.43, *p* = .004). For individual tracts, significant additional variance contributed by the mean and variability of depressive symptoms was found in the middle cerebellar peduncle (*F*_2,14686_ = 4.63, *p* = .010).

### Associations Between Measures of Depressive Symptoms and Behavioral Traits

The cross-sectional measure of depressive symptoms was positively and significantly correlated with all 3 longitudinal measures of depressive symptoms with *r*s = .457, .840, and .478 (all *p*s < 10^−16^) for the correlations with longitudinal slope, mean, and variability of depressive symptoms, respectively. A correlation matrix between the measures can be found in Supplemental Table S1.

For the depression-related behavioral variables, contrary to the imaging results, the longitudinal trajectory of symptoms over time had the lowest measures of associations (absolute βs = .026–.081, *p*_corr_ < .043 for significant associations) compared with the other 3 measures of depressive symptoms. The largest effects were for overall mean symptom severity (absolute βs = .048–.216, *p*_corr_ < 3.81 × 10^−6^). There was no clear pattern of difference between associations with current symptoms and the longitudinal mean and variability for these behavioral variables. There were, however, indications of a similar pattern of the strongest associations for all 3 measures of depression severity with neuroticism, insomnia, and pain (cross-sectional: absolute βs = .195–.524, *p*_corr_ < 1 × 10^−30^; mean: absolute βs = .216–.567, *p*_corr_ < 1 × 10^−30^; variability: absolute βs = .145–.399, *p*_corr_ < 1 × 10^−30^). Other significant associations are detailed in Table 1.

**Table 1:** Associations Between Measures of Depressive Symptoms and Behavioral Variables

Category	Dependent Variable	Factor							
		Cross-sectional		Longitudinal Slope		Mean		Variability	
		Beta/Log(OR)	*p*_corr_	Beta/Log(OR)	*p*_corr_	Beta/Log(OR)	*p*_corr_	Beta/Log(OR)	*p*_corr_
Mental Health	Neuroticism	.524^a^	<1 × 10^−30^^a^	.081^a^	3.17 × 10^−7^^a^	.567^a^^*,*^^b^	<1 × 10^−30^^a^	.399^a^	<1 × 10^−30^^a^
	Age of onset for depression	−.056^a^	2.39 × 10^−8^^a^	−.048	.052	−.065^a^^*,*^^b^	2.05 × 10^−10^^a^	−.061^a^	2.10 × 10^−9^^a^
Sociodemographics	Household income	−.11^a^	<1 × 10^−30^^a^	.035	.056	−.142^a^^*,*^^b^	<1 × 10^−30^^a^	−.125^a^	<1 × 10^−30^^a^
	Education	.057	.095	−.054	.604	.054	.118	.002	.953
	Townsend index tertile	−.058^a^	7.04 × 10^−11^^a^	−8.48 × 10^−4^	.970	−.079^a^^*,*^^b^	2.17 × 10^−19^^a^	−.049^a^	1.20 × 10^−8^^a^
Lifestyle Measures	Insomnia	.207^a^	<1 × 10^−30^^a^	.017	.424	.222^a^^*,*^^b^	<1 × 10^−30^^a^	.154^a^	<1 × 10^−30^^a^
	Smoking status	.056^a^	4.34 × 10^−14^^a^	5.50 × 10^−4^	.970	.075^a^^*,*^^b^	1.03 × 10^−23^^a^	.05^a^	1.35 × 10^−9^^a^
Physical Measures	Body mass index	.097^a^	<1 × 10^−30^^a^	−.014	.520	.129^a^^*,*^^b^	<1 × 10^−30^^a^	.096^a^	<1 × 10^−30^^a^
	Ever pain last month	.195^a^	<1 × 10^−30^^a^	.01	.604	.216^a^^*,*^^b^	<1 × 10^−30^^a^	.145^a^	<1 × 10^−30^^a^
	Hand grip strength	−.039^a^	5.68 × 10^−16^^a^	.026^a^	.043^a^	−.048^a^^,^^b^	3.20 × 10^−23^^a^	−.034^a^	1.32 × 10^−10^^a^
Cognition	g.Cognition (total)	−.043^a^	.001^a^	.045	.305	−.063^a^	3.81 × 10^−6^^a^	−.081^a^^*,*^^b^	1.28 × 10^−7^^a^
	g.Cognition (processing speed)	−.058^a^	9.78 × 10^−8^^a^	.036	.305	−.077^a^	3.54 × 10^−12^^a^	−.084^a^^*,*^^b^	7.44 × 10^−12^^a^

g.Cognition, general variance of cognitive tests; OR, odds ratio.

a Items that showed significant associations with any of the measures for depressive symptoms.

b The greatest effect sizes across all 4 measures for each line.

## Discussion

In the current investigation, we report novel patterns of association between 4 measures of cross-sectional and longitudinal depressive symptom severity with decreased white matter microstructural integrity. Decreased white matter microstructure in the anterior thalamic radiation demonstrated significant associations across all 4 measures of depressive symptoms (for MD: βs = .028–.030). The strongest white matter associations were found for variables relating to increasing longitudinal symptom severity. Measures of current symptom severity (cross-sectional measures) were particularly associated with decreased white matter integrity in association fibers and thalamic radiations (for MD: βs = .015–.039). Associations with higher mean and variability of depressive symptoms over time, however, showed associations primarily in projection fibers (for MD: βs = .019–.029). Contrary to the imaging findings, the nonimaging variables, in particular neuroticism, insomnia, and pain, were associated with mean and variability of symptoms over time, as well as with current symptoms rather than with longitudinal change in symptoms.

Stable and transient conditions, referred to as trait and state in other research contexts, are two related yet distinctive features contributing to individual differences in mood conditions. Recent population-based genetic and predictive modeling studies have revealed that stable manifestations of emotional problems typically have a higher heritability than more transient features (36). In addition, longitudinal measures, including variability, had a higher predictive power relating to severe forms of behavior such as suicide attempts (37). Hence, understanding the biological basis of these potentially distinctive features would have important implications 38, 39.

Lower microstructural integrity in anterior thalamic radiation in particular showed associations with all 4 measures of depressive symptoms. The thalamic radiation tract subset was also consistently associated with all measures of depressive symptoms (nominally significant for the variability measure and significant for all other measures). This shared mechanism between measures indicates the importance of the thalamic system, particularly frontothalamic connectivity, in depressive symptomatology. Our results could therefore explain why thalamic radiations are one of the most replicated findings in either lifetime or cross-sectional case-control studies of MDD 16, 40, 41. First, lower microstructural integrity of the thalamic radiation may be particularly susceptible to the influence by early life factors 42, 43 and, second, they may relate to genetic predisposition to depression (17). Although the observational data in the current study did not allow for causal inferences, in future studies thalamic radiations may be a strong candidate as a causal biomarker for illness.

Associations between cross-sectional measures with fibers in the subset of association fibers were particularly significant after correcting for longitudinal measures, indicating that these regions are particularly sensitive to temporary variations. Lower microstructural integrity in association fibers was also associated with worsening depressive symptoms over time. Association fibers and connections to the prefrontal cortex have been repeatedly found to be associated with executive cognition 44, 45, 46 and closely related to psychological resilience (47). Abnormalities in these tracts could therefore contribute to both temporary depressive status and longitudinal decline in mental well-being (48).

Projection fibers were particularly associated with mean and variability of depressive symptoms. Microstructure in this white matter subset is well known for being related to motor response and processing speed. Therefore, the results suggest that higher mean and variability of depressive symptoms may be related to cognitive decline and decline of psychomotor abilities 49, 50.

We note importantly that most of the current results were found for MD rather than FA. Despite the differences in level of significance, MD and FA presented similar directions of effects, and the scales of effect were similar for the most robust findings, especially in thalamic radiations (Supplemental Table S3). The discordance between MD and FA may be rooted in the differential sensitivity of MD and FA to a variety of complex degenerative processes. Changes in FA, which could result, for example, from increased transverse diffusion that is due to myelin and axonal disruption (51), may be masked by co-occurring processes such as fiber reorganization and glial reactivity. In such instances, where all 3 eigenvectors of the diffusion tensor experience proportional change, it is plausible that MD would offer greater sensitivity (52). Notably, MD also reportedly exhibits greater sensitivity than FA to other traits such as aging (44).

In the current study, we used a very large imaging sample. Although the sample size of longitudinal trajectory was much lower, it was still much larger than that in most neuroimaging studies, especially considering that the data are longitudinal and cover up to 10 years. All this provides high statistical power to reliably detect modest associations (53). However, a limitation of the current study is that the time lag between adjacent assessments may vary per participant from 3 to 6 years. For this reason, we also adjusted for this difference by controlling for the age of each time point. Another limitation is that there is a known healthy volunteer bias within the UK Biobank sample (54) as a result of the large-scale, population-based recruitment strategy. Variation of effect sizes may be expected in other samples of in-hospital patients. Finally, we note that it is possible that some of the behavioral patterns associated with longitudinal variation of depressive symptoms may be able to partially explain the neurobiological associations. Future work is needed to identify intermediate variables that mediate the association between brain structural measures and longitudinal progression of depressive symptoms.

Our results provide evidence based on a large-scale imaging dataset that white matter microstructure is related to slope of longitudinal trajectory of depressive symptoms, current symptom severity, and mean and variability of depressive symptoms, with overlapping regional associations in MD in thalamic radiations as well as distinctive regional patterns between longitudinal mean and variability measures and current symptoms. Further mechanistic insights underlying the relationship between changes in neurobiology and changing symptoms will be dependent on availability of future large-scale longitudinal neuroimaging datasets along with availability of methods and tools able to test for causal inferences.

## Supplementary Material

Key Resources Table
