# Supplementary material for: White Matter Microstructure and Its Relation to Longitudinal Measures of Depressive Symptoms in Mid- and Late Life
Source: Biol Psychiatry. 2019 Nov 15;86(10):759–68. doi: 10.1016/j.biopsych.2019.06.011 (PMC6906887; doi:10.1016/j.biopsych.2019.06.011)
Supplement: Supplemental Material [file mmc2.pdf]

White Matter Microstructure and Its Relation to Longitudinal Measures of Depressive Symptoms in Mid-late Life

Supplementary Information

Table of Contents

Supplementary Methods

Supplementary Results

Figures S1-S7

Tables S1-S8

Supplementary References

Supplementary Methods

PHQ-4 questionnaire

PHQ-4 (Patient Health Questionaire-4) questions include: "Frequency of depressed mood in last 2 weeks", "Frequency of unenthusiasm/disinterest in last 2 weeks", "Frequency of tenseness/restlessness in last 2 weeks" and "Frequency of tiredness/lethargy in last 2 weeks". This questionnaire assesses depression-related symptoms within a 2-week timeframe. The sum of the scores was calculated to indicate depressive symptoms.

The mean time lag between the first and second occasion was 4.29 years with a standard deviation of 0.94 years. Between the second and third occasion, mean time lag was 3.32 years with a standard deviation of 1.19 years. Between the third and the final occasion, mean time lag was 0.003 years with a standard deviation of 1.10 years.

For each measure of depressive symptoms derived from cross-sectional assessments, the mean time lag of mean level of depressive symptoms was 8.04 years (sd=1.22 years), the variability of depressive symptoms was 8.09 years (sd=1.14 years), and slope of longitudinal trajectory was 8.26 years (sd=1.11 years). The correlations between longitudinal measures and time lag was very small and therefore this variable was not included in the main model (r ranged from 2.41×10^{-4} to 0.027).

dMRI measures

All imaging data was acquired using a 3T Siemens Skyra (software platform VD13) machine, using a standard ("monopolar") Stejskal-Tanner pulse sequence. FSL packages were used for data pre-processing and microstructure estimation (1). Pre-processing included correction for eddy

currents, head-motion, and gradient distortion, using the Eddy tool (2). The processed tracts included 12 bilateral tracts that has a value for each brain hemisphere (acoustic radiation, anterior thalamic radiation, cingulate gyrus part of cingulum, corticospinal tract, inferior fronto-occipital fasciculus, inferior longitudinal fasciculus, medial lemniscus, parahippocampal part of cingulum, posterior thalamic radiation, superior longitudinal fasciculus, superior thalamic radiation and uncinate fasciculus) and 3 unilateral tracts (forceps major, forceps minor and middle cerebellar peduncle).

Depressive symptoms

For the growth curve model, we used the 'growth' function from the 'lavaan' package (http://lavaan.ugent.be/tutorial/growth.html) in R (3). Scaled age at each assessment was controlled for. The growth curve model showed good fit to the data (CFI = 0.988, TLI = 0.984, RMSEA = 0.031, SRMR = 0.014, Chi-square (17) = 103.563 with a $p<0.001$). Longitudinal change within the whole population was in a negative direction but did not reach to significance (β = -0.094, p = 0.164). Both the intercept (β= 7.366, $p < 0.001$) and variance (β=1.521, $p < 0.001$) of the mean slope of growth curve model were significant. Each individual's slope of longitudinal trajectory was estimated for further analysis by using the 'predict' function in 'lavaan'.

Covariates

In addition to age, age^2 and gender, we also included scanner positions for all three axis, alcohol consumption, smoking status and stressful life events. The covariates except for age and gender

will be explained in detail below. All the covariates described here were acquired with the imaging assessments. See also Table S1, S6 and S7.

The scanner position was used for controlling for systematic change in the static magnetic field (the last four fields in http://biobank.ctsu.ox.ac.uk/crystal/label.cgi?id=110). These proxies for scanner position showed minimal correlation with our white matter phenotypes, but in order to achieve a better estimated model, we chose to include them in our models.

Alcohol consumption was self-reported weekly consumption which was used in a published paper on the overall UK Biobank sample of about 500k people (4). We used a slightly different approach to exclude impossible numbers. In the referenced study, they excluded values over 5 standard deviations from mean, and we employed their values as upper and low thresholds instead of calculating our own standard deviations and mean in the subsample with imaging data, because we have a much smaller sample, which may introduce more noise and exclude an excessive amount of people.

For smoking status, we used the self-reported smoking status information (http://biobank.ctsu.ox.ac.uk/crystal/field.cgi?id=20116). Participants could choose from one of the four options: (a) current smoker, (b) previous smoker, (c) non-smoker and (d) prefer not to answer. There were NAs for those did not answer. As the number of people who chose ‘prefer not to answer’ was very small, we did not transfer this into NA so to maximize our sample size. We treated this covariate as a categorical variable in our model. For the sensitivity analysis shown in Table S6 and S7, in order to make it easier for demonstration, we transferred it into a numeric variable (current smoker = 2, previous smoker =1, non-smoker = 0, prefer not to answer = NA). This still represents the effects of smoking to depressive symptoms and white matter

microstructure, as it is generally believed that there should be a gradient effect from current smoker to non-smoker.

Stressful life events described the number of events happened within 2 years before scanning session (http://biobank.ctsu.ox.ac.uk/crystal/field.cgi?id=6145). Items include: serious illness, injury or assault to oneself, death of a close relative, death of a spouse or partner, marital separation/divorce and financial difficulties.

Other behavioural measures included in the present study

All the behavioural variables were collected along with the imaging assessment unless it was collected within other categories such as online-follow up questionnaires that were not collected at any of the UK Biobank assessment centres.

Variables collected with the imaging assessment are listed below (relevant URL in the data showcase website shown in the brackets). For all the answers for the items listed below, “Prefer not to answer” and “Do not know” were recoded as NA.

1. Insomnia (http://biobank.ctsu.ox.ac.uk/showcase/field.cgi?id=1200), as indicated by 1=Never/rarely, 2=Sometimes, and 3=Usually.
2. Smoking status (http://biobank.ctsu.ox.ac.uk/showcase/field.cgi?id=20116), with 0=Never, 1=Previous, and 2=Current.
3. Recent pains in last month (http://biobank.ctsu.ox.ac.uk/showcase/field.cgi?id=6159), with 1=any type of pain reported and 0=None of the above. This variable was set as binary and logistic regression was used for its association tests. Therefore log odds ratios were reported (see Table 1).

4. Hand grip strength was derived as the mean of left- and right-hand grip strength (http://biobank.ctsu.ox.ac.uk/showcase/field.cgi?id=46, http://biobank.ctsu.ox.ac.uk/showcase/field.cgi?id=47).
5. g.Cognition. A selection of variables for cognitive functions was included to generate a g factor that represents the variance of general cognitive ability. Cognitive tasks include: Reaction time (http://biobank.ctsu.ox.ac.uk/showcase/field.cgi?id=20023), Verbal-numeric reasoning (also referred to as fluid intelligence in UK Biobank data dictionary: http://biobank.ctsu.ox.ac.uk/showcase/field.cgi?id=20016), Trail making (derived by Trail B – Trail A: http://biobank.ctsu.ox.ac.uk/showcase/field.cgi?id=6350, http://biobank.ctsu.ox.ac.uk/showcase/field.cgi?id=6348), Pair matching (http://biobank.ctsu.ox.ac.uk/showcase/field.cgi?id=399), Digit symbol substitution (http://biobank.ctsu.ox.ac.uk/showcase/field.cgi?id=23324), Matrix pattern completion (http://biobank.ctsu.ox.ac.uk/showcase/field.cgi?id=6373) and Tower rearranging (http://biobank.ctsu.ox.ac.uk/showcase/field.cgi?id=21004). A principal component analysis (PCA) was performed and the score of the first unrotated principal component was extracted as a measure of g factor for cognitive functions. The first component explained 34.5% of the total variance. Absolute correlation loadings for each item ranged from 0.350 to 0.691 (loadings for reaction time and pair matching were negative, as for these items a smaller reaction time is better).
6. g.Cognition for processing speed. An independent PCA was conducted on a selected range of the above tasks that has explicated stated measuring processing speed. The tasks included were: Reaction time, Trail making which tests visual processing speed, Digit symbol substitution which tests complex processing speed, and Pair matching, Matrix pattern completion, Tower rearranging and Verbal-numeric reasoning as participants were instructed to give answers in a limited time frame. Similar as g.Cognition, these measures used the score of the first unrotated principal component. The first principal component explained 42.2% of total variance. Absolute correlation loadings ranged from 0.553 to 0.734 (loading for reaction time was negative and all the others positive).

7. Neuroticism. Fields from 1920 to 2030 in the URL http://biobank.ctsu.ox.ac.uk/showcase/label.cgi?id=100060 were used, and a total number of items with a 'Yes' answer for each participant was calculated as the neuroticism score.

Other variables that were not acquired with the imaging assessment were also often considered as lifetime variables that largely remain stable over time. These include the age of onset for depression (http://biobank.ctsu.ox.ac.uk/showcase/field.cgi?id=20433), household income (http://biobank.ctsu.ox.ac.uk/showcase/field.cgi?id=738), educational attainment (http://biobank.ctsu.ox.ac.uk/showcase/field.cgi?id=6138, 1=College or University degree, 0=others that are less than College or University degree), Townsend Index (http://biobank.ctsu.ox.ac.uk/showcase/field.cgi?id=189, 1=the least deprived tertile, 2=the second tertile, 3=the most deprived tertile).

Supplementary Results

Associations between measures of depressive symptoms and NODDI measures

In general, results for ISOVF showed the largest resemblance with results for MD (see Figure S3). Here we report the results for all NODDI measures for each measure of depressive symptoms.

Cross-sectional assessment of depressive symptoms

For general variances of NODDI measures, higher cross-sectional depressive symptoms were associated with higher global ISOVF, higher ISOVF in gAF, gTR and gPF (β ranged from 0.018 to 0.029, p_{corr} ranged from 0.012 to 1.83×10^{-5}). Higher cross-sectional measure was also associated

with lower OD in gTR (β = -0.016, p_{corr} = 0.047).

Tract-specific associations were mainly found in ISOVF. Higher cross-sectional depressive symptoms was associated with higher ISOVF in anterior thalamic radiations, cingulate gyrus part of cingulum, parahippocampal part of cingulum, corticospinal tract, inferior fronto-occipital fasciculus, inferior longitudinal fasciculus, superior longitudinal fasciculus, superior thalamic radiation, uncinate fasciculus and middle cerebellar peduncle (β ranged from 0.015 to 0.039, p_{corr} ranged from 0.048 to 6.45×10^{-8}). For other NODDI measures, higher cross-sectional measure was positively associated with OD in medial lemniscus (β = 0.018, p_{corr} = 0.050) and negatively associated with OD in superior thalamic radiation (β = -0.018, p_{corr} = 0.050).

No general or tract-specific associations were found for ICVF (p_{corr}>0.314).

Longitudinal trajectory of depressive symptoms

For general variance in NODDI measures, a higher slope of longitudinal increase of depressive symptoms was associated with lower OD in global variance and gPF (β ranged from-0.035 to -0.039, p_{corr} ranged from 0.046 to 0.045). No general variance in ISOVF (p_{corr}>0.052) or ICVF (p_{corr}>0.580) showed a significant association with longitudinal slope.

For specific tracts, a steeper slope of longitudinal trajectory was associated with higher ISOVF in corticospinal tract and superior thalamic radiation (β ranged from 0.029 to 0.036, p_{corr} ranged from 0.040 to 0.025), as well as lower OD in inferior longitudinal fasciculus (β = -0.051, p_{corr} = 0.004). No tract measure was found associated with the slope of the longitudinal growth curve for ICVF measures (all p_{corr} > 0.314).

The mean of depressive symptoms derived from multiple assessments

Associations regarding mean depressive symptoms were mainly shown in ISOVF. Higher mean depressive symptoms were associated with higher ISOVF in global variance, gAF, gTR and gPF (β ranged from 0.014 to 0.027, p_{corr} ranged from 0.046 to 3.29×10^{-4}). It is also associated with higher gAF OD (β = 0.016, p_{corr} = 0.047) and lower gTR OD (β = -0.016, p_{corr} = 0.049). Tract-wise analysis showed that ISOVF of anterior thalamic radiations, cingulate gyrus part of cingulum, corticospinal tract, superior longitudinal fasciculus, superior thalamic radiation, uncinate fasciculus and middle cerebellar peduncle (β ranged from 0.017 to 0.029, p_{corr} ranged from 0.021 to 5.32×10^{-9}) were associated with higher mean depressive symptoms. Other than the associations with mean depressive symptoms shown in ISOVF, other association was found in lower OD in parahippocampal part of cingulum (β = 0.016, p_{corr} = 0.050), medial lemniscus (β = 0.030, p_{corr} = 6.83×10^{-4}), and superior thalamic radiation (β = -0.019, p_{corr} = 0.050).

The variability of depressive symptoms derived from multiple assessments

For general variance, positive associations were shown in all g measures including global variance, gAF, gTR and gPF in ISOVF (β ranged from 0.016 to 0.033, p_{corr} ranged from 0.042 to 1.83×10^{-4}). Negative associations in OD were found in global variance and gAF (β ranged from 0.019 to 0.023, p_{corr} ranged from 0.046 to 0.040).

Tract-wise analysis showed that variability of depressive symptoms were positively associated with ISOVF in anterior thalamic radiations, superior thalamic radiation, uncinate fasciculus and middle cerebellar peduncle (β ranged from 0.015 to 0.037, p_{corr} ranged from 0.048 to 5.64×10^{-6}). Other than measures of ISOVF, other association was found between variability of depressive symptoms and OD in parahippocampal part of cingulum and medial lemniscus (β ranged from 0.020 to 0.021,

p_{corr} ranged from 0.047 to 0.050).

For the above associations, we ran additional models to test if the results would change when smoking status, SLE and alcohol consumption were not corrected for. No association found in the main model where the three covariates were included turned insignificant after the covariates were removed (see Figure S5).

Effect of partial-volume contamination to FA and MD

We observed differences of results for FA and MD. A possible explanation may be the different effect of partial volume contamination on FA and MD. To control for possible effects of partial-volume contamination related to structural atrophy, we have included age and age^2 as covariates(5). We also conducted an additional analysis including brain size (in UK Biobank data dictionary: f.25010.2.0, described as "Volume of Brain, grey+white matter") as one of the covariates and the results remained significant for both FA and MD except for one association turned null for MD in anterior thalamic radiation after controlling for brain size, however reached nominal significance before multiple correction applied (β = 0.030, p_{uncorr} = 0.020, p_{corr} = 0.053) (Figure S6).

Effect of recent antidepressant intake on white matter microstructure

We have conducted a sensitivity analysis, removing participants who had medication only around the imaging assessment but not on other occasions. This was to test if removing the effect of recent intake of antidepressants would change the results.

First, we extracted a list of drug names from the British National Formulary-70 (BNF 70) (https://www.bnf.org/products/books/) under the category for depression. Drug names that

matched the entries in treatment/medication (field name: f.20003, URL: https://biobank.ctsu.ox.ac.uk/crystal/field.cgi?id=20003) were identified as reported antidepressant intake. For the last instance of online follow-up questionnaires, the information of antidepressant intake was assessed in a different way by asking whether there is any substance taken for depression (field name: f.20546, URL: http://biobank.ctsu.ox.ac.uk/showcase/field.cgi?id=20546). Answers of “medication prescribed to you (for at least two weeks)” were identified as antidepressant intakes.

Participants who reported intake of antidepressant only at the imaging assessment (instance = 2), but not on other occasions (instance = 0/1/3) were removed from analysis, in order to remove the effect of antidepressant taken recently. Data from a total of 323 people were removed from analysis, and 19,345 remained.

The same regression models were conducted on this subsample, results are shown in Figure S7. Regional patterns for this subsample are very similar with the main findings (in main text).

Figure S1. Description of sample sizes and changes due to each step of data merging or outlier removal. Cross-sectional = Cross-sectional measure at the imaging assessment, Mean = mean level of depressive symptoms based on multiple assessments for at least two times, Variability = standard deviation of depressive level of multiple assessments for at least three times, and Longitudinal slope = slope of longitudinal changes over all four times of assessments.

Recruited
N=22,346

↓

Scanned
N=22,266

↓

Included in IDP dataset
(non-pilot, passed quality check)
N=19,345

↓

Outliers excluded
N_{FA}=19,252 N_{MD}=19,207
N_{ICVF}=19,262 N_{ISOVF}=19,145 N_{OD}=19,215

↓

Merged with distress measures

	FA	MD	ICVF	ISOVF	OD
**Cross-sectional measure**	19,025	18,979	19,032	18,916	18,984
**Longitudinal slope**	4,457	4,449	4,454	4,436	4,448
**Mean**	19,133	19,087	19,142	19,028	19,095
**Variability**	14,813	14,782	14,818	14,743	14,786

↓

Merged with covariates

	FA	MD	ICVF	ISOVF	OD
**Cross-sectional measure**	18,941	18,897	18,948	18,833	18,904
**Longitudinal slope**	4,444	4,436	4,441	4,423	4,435
**Mean**	18,951	18,906	18,959	18,846	18,915
**Variability**	14,739	14,708	14,743	14,669	14,713

Figure S2. Illustration of WM tracts. The tracts were defined by tractography mapping on FA (fractional anisotropy) data using AutoPtx (https://fsl.fmrib.ox.ac.uk/fsl/fslwiki/AutoPtx). They were categorised into three subsets as shown in the figure. Forceps major, forceps minor and uncinate fasciculus are unilateral structures and the rest are bilateral. For the purpose of clear illustration, bilateral structures were shown identically in both hemispheres.

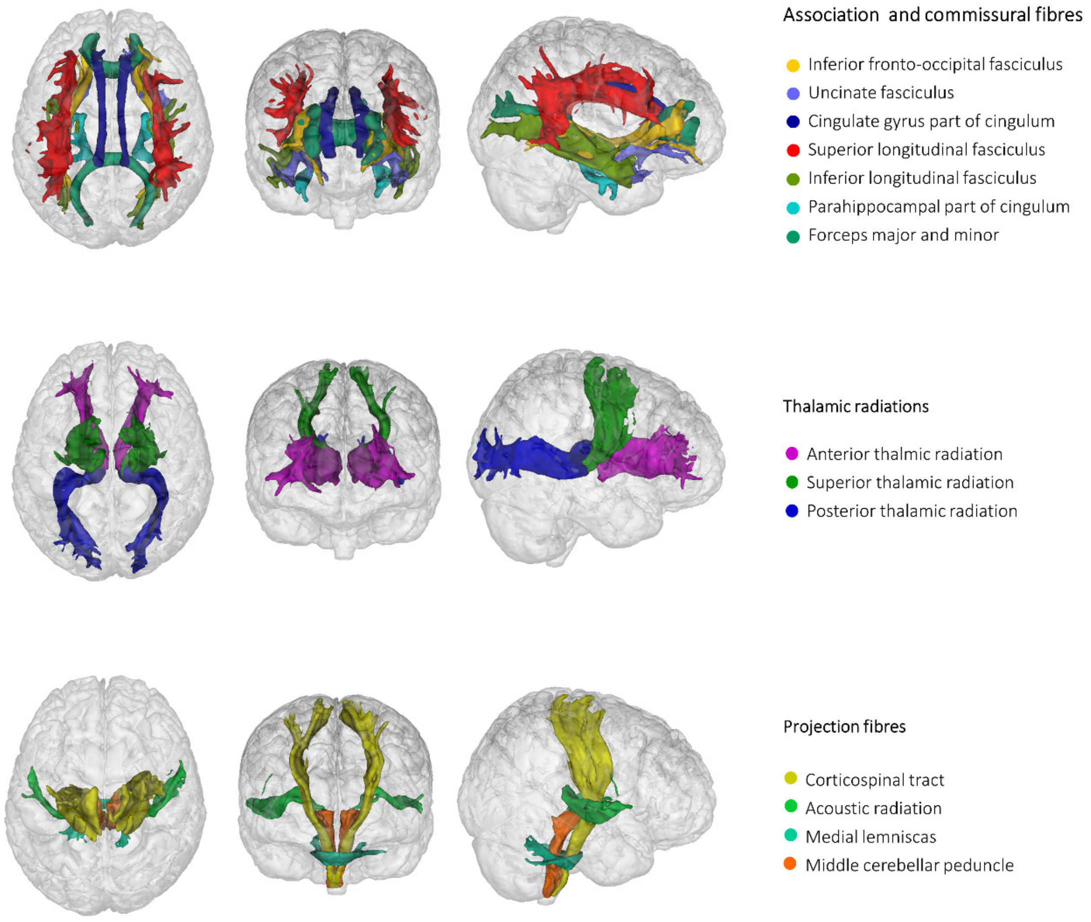

Figure S3. Results for all dMRI measures including DTI and NODDI. More descriptive statistics were shown in Table S3 and S8, results in the main text and supplementary results. In the heatmaps, each colour theme represents one dMRI measure. For the measures of depressive symptoms: Cross-sectional = depressive level at the imaging assessment, Longitudinal slope = slope of longitudinal changes over three time points until the imaging assessment, Mean = mean level of depressive symptoms based on multiple assessments for at least two times, Variability = standard deviation of depressive level of multiple assessments for at least three times. As the measures of FA, ICVF and OD have negative direction with MD and ISOVF, here in this figure, the effect sizes for FA, ICVF and OD were reversed (×-1). Significant associations after FDR correction were marked with a single asterisk.

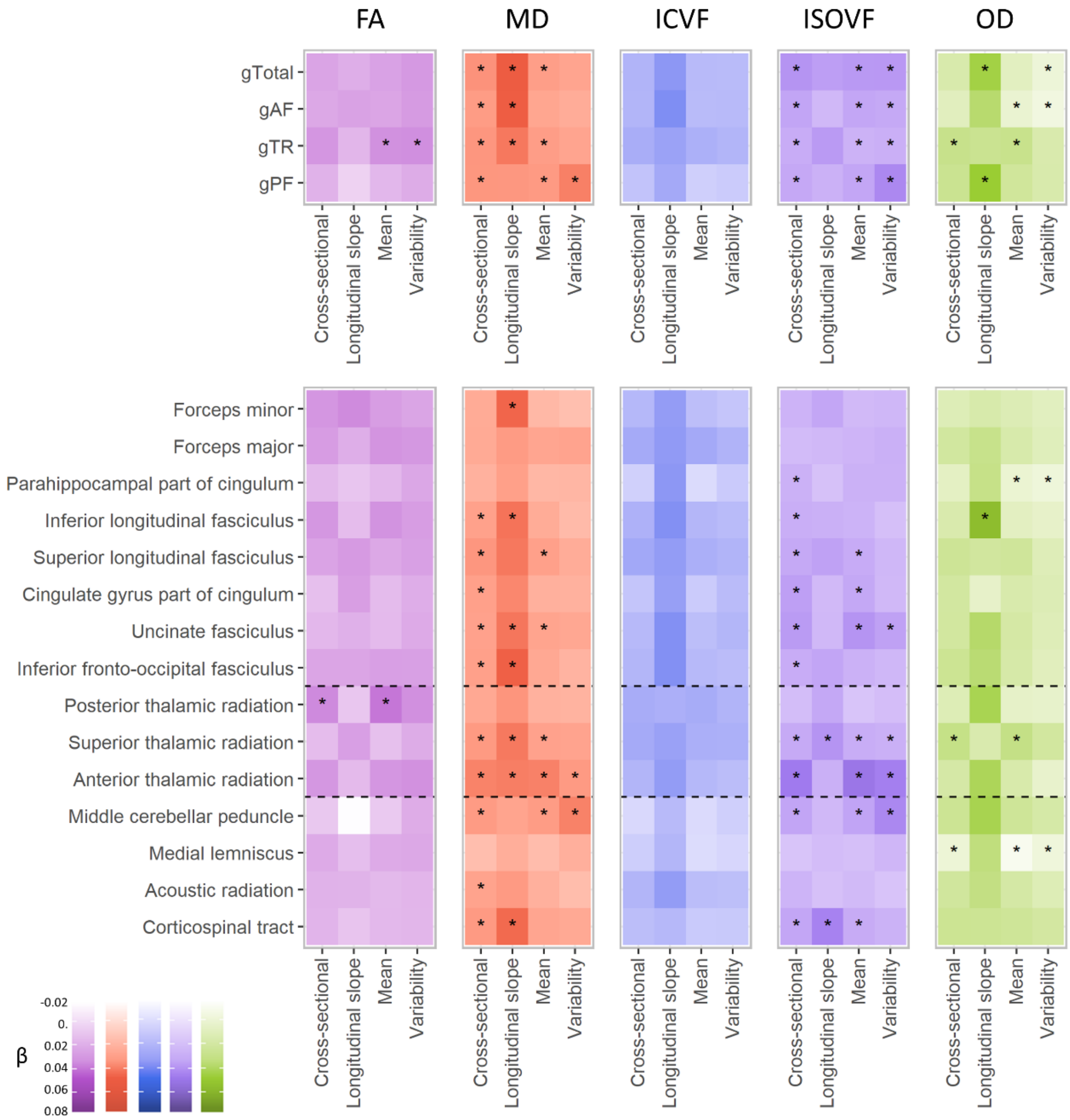

Figure S4. Variance explained by the principal components of PCA on total and subsets of white matter tracts.

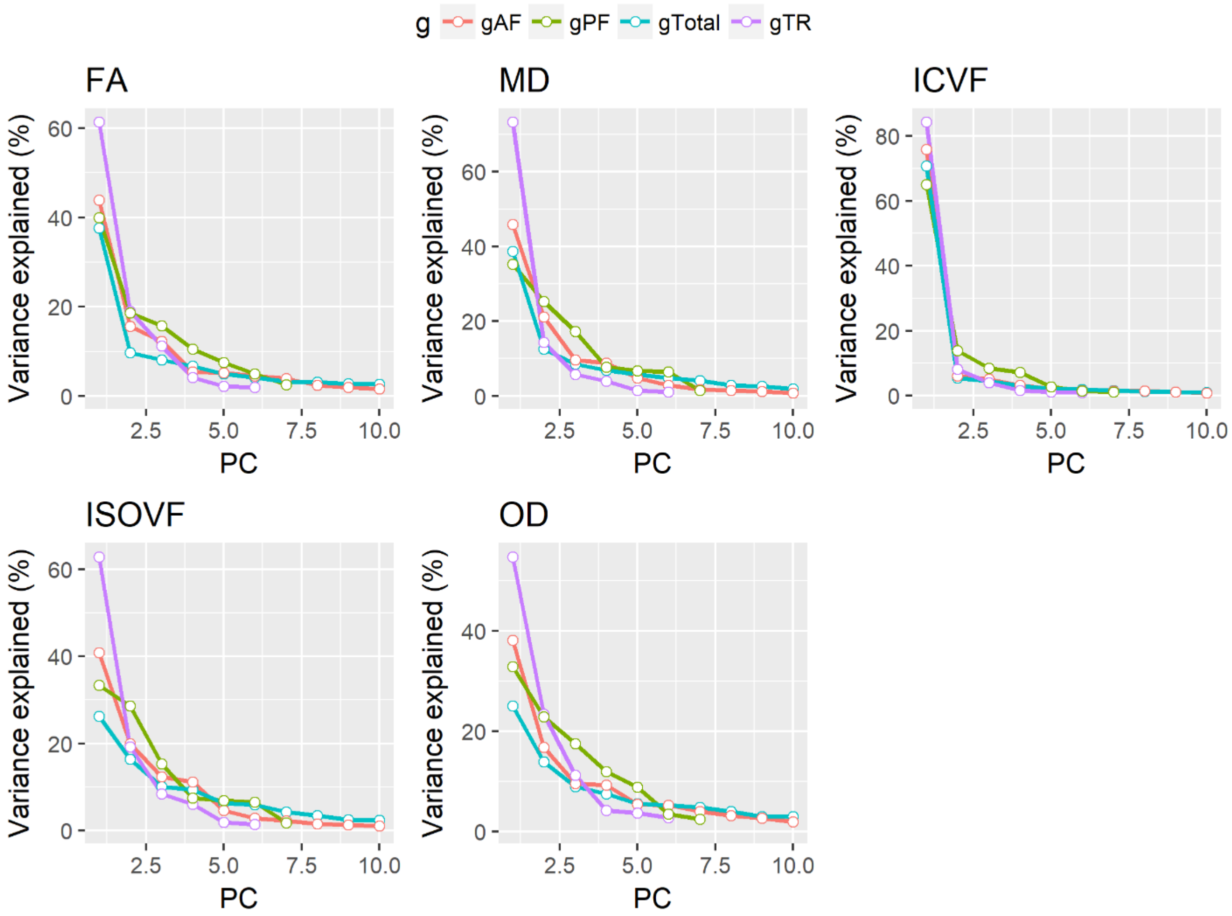

Figure S5. Results for a secondary model without controlling for stressful life events, smoking status or alcohol consumption. For the abbreviations and multiple correction methods, see the legend of Figure S3.

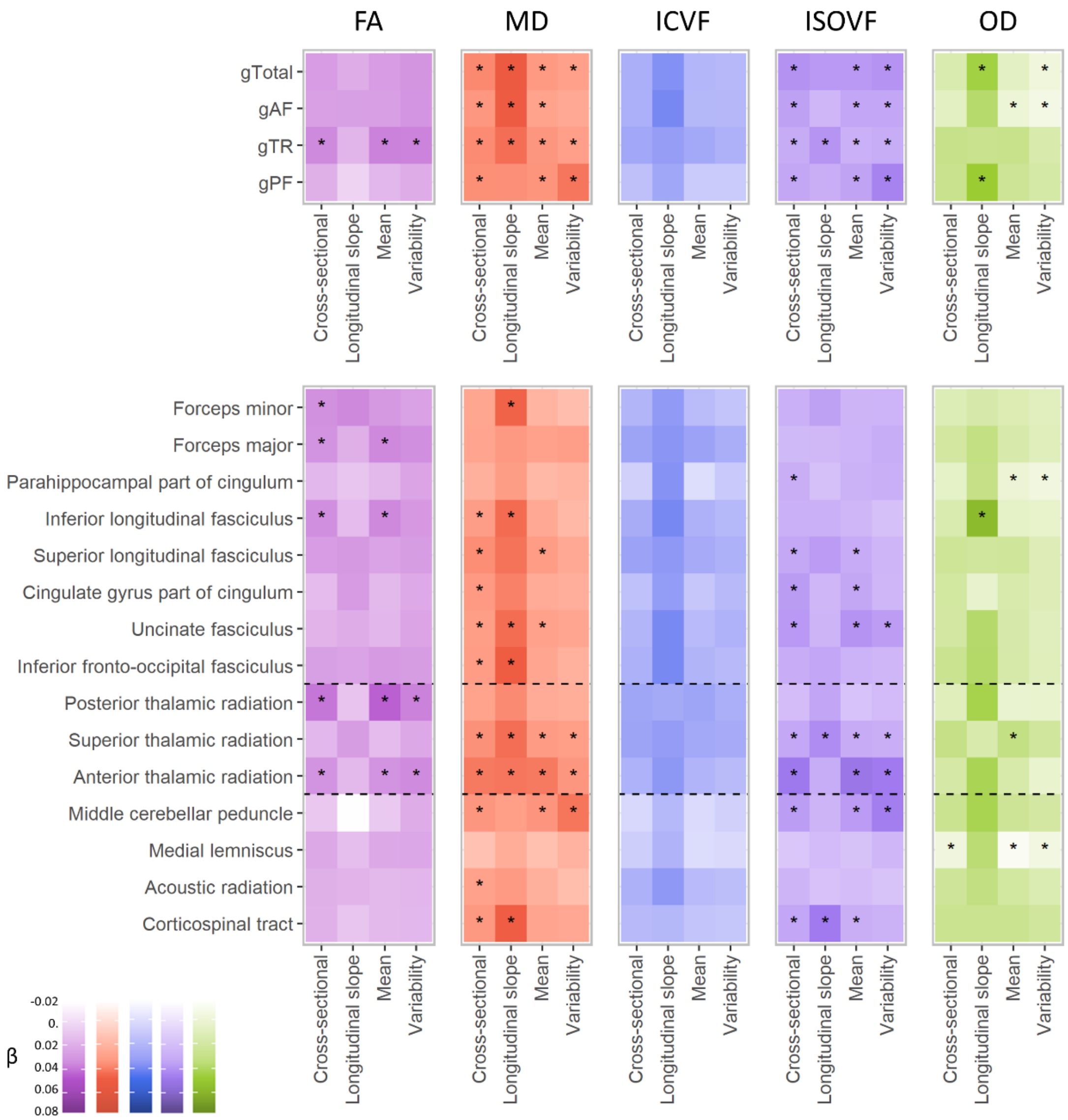

Figure S6. Results for adding brain size as a covariate.

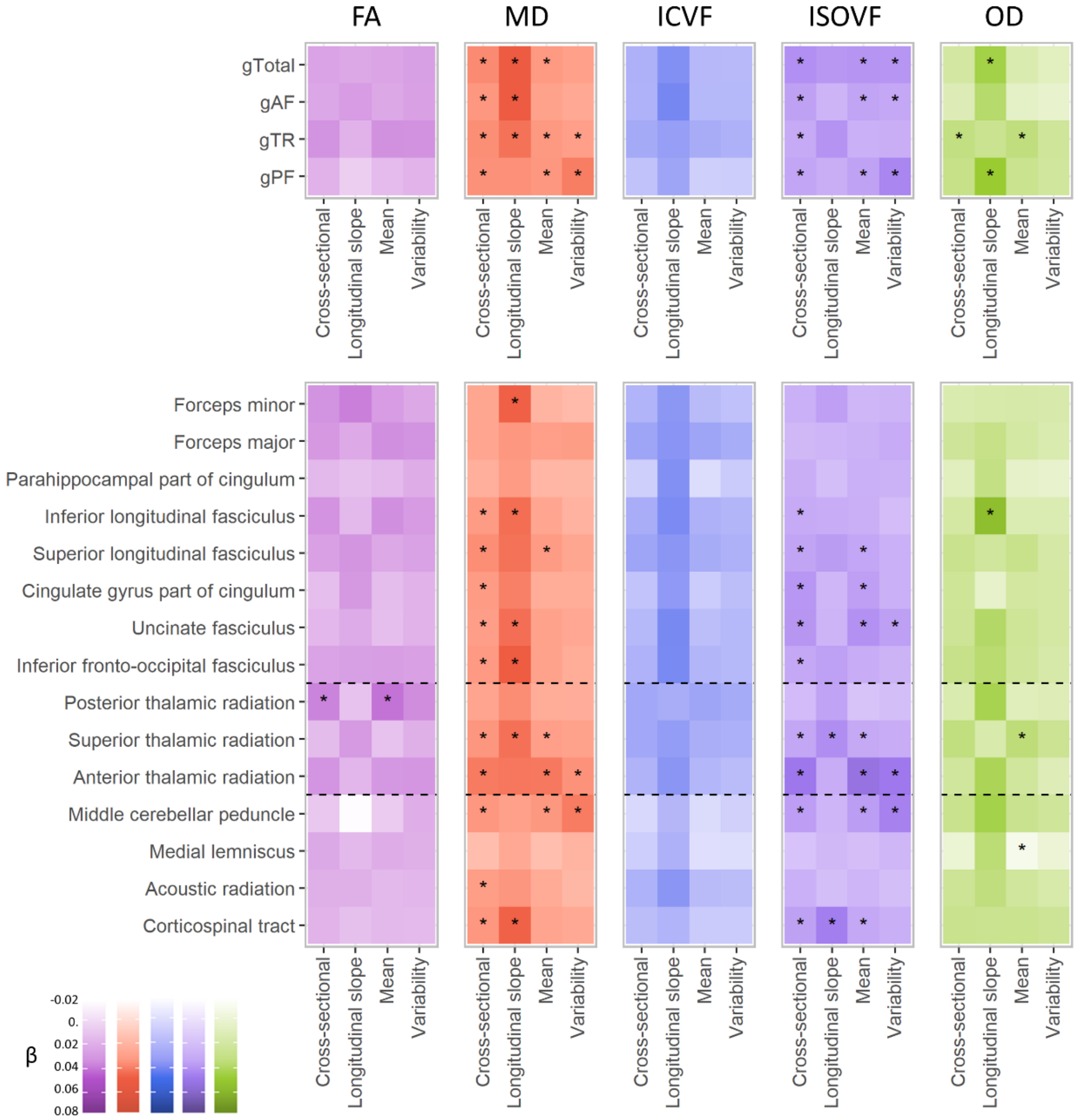

Figure S7. Results for removing data from participants who had recent medication intake around the imaging assessment.

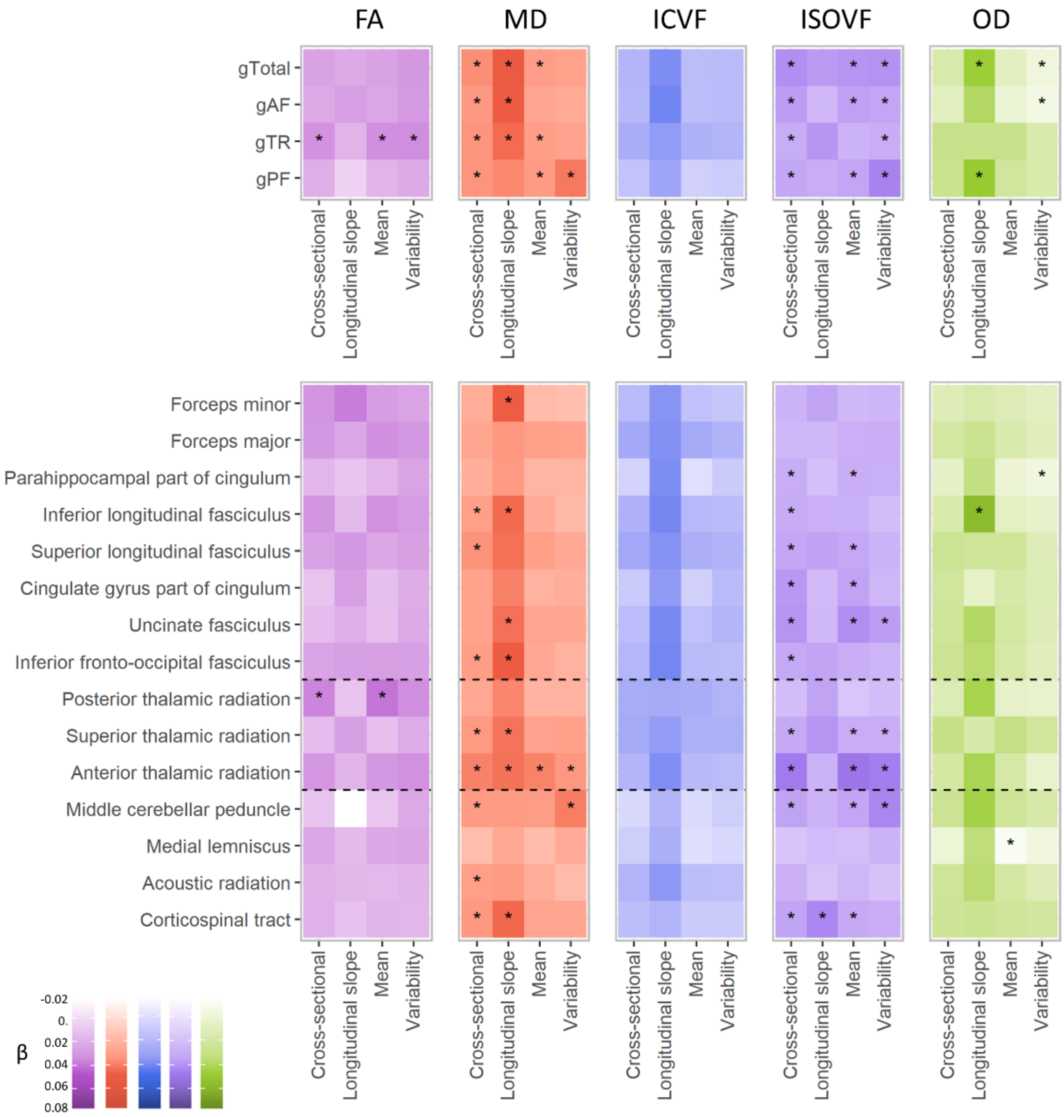

Table S1. Correlation matrix for all measures for depressive symptoms, smoking status, alcohol consumption, stressful life events, neuroticism and age range (time lag) for the multiple assessments used for generating mean level of depressive symptoms. For the measures of depressive symptoms: Cross-sectional measure = depressive level at the imaging assessment, Mean = mean level of depressive symptoms based on multiple assessments for at least two times, Variability = standard deviation of depressive level of multiple assessments for at least three times, and Longitudinal slope = slope of longitudinal changes over all four times of assessments. All r>0.3 are highlighted in bold. Measures for depressive symptoms were correlated with one another.

	Cross-sectional measure	Mean	Variability	Longitudinal slope	Smoking	Alcohol	SLE	N of occasions	Time.lag
Cross-sectional measure	1	**0.84**	**0.478**	**0.457**	0.04	-0.035	0.18	--	--
Mean	**0.84**	1	**0.651**	-0.046	0.055	-0.037	0.189	-0.053	0.013
Variability	**0.478**	**0.651**	1	-0.124	0.037	-0.024	0.127	0.03	0.014
Longitudinal slope	**0.457**	-0.046	-0.124	1	-0.002	0.005	0.041	--	0.028
Smoking	0.04	0.055	0.037	-0.002	1	0.2	0.005	-0.013	-0.011
Alcohol	-0.035	-0.037	-0.024	0.005	0.2	1	-0.036	0.001	0
SLE	0.18	0.189	0.127	0.041	0.005	-0.036	1	-0.002	-0.008
N of occasions	--	-0.053	0.03	--	-0.013	0.001	-0.002	1	--
Time.lag	--	0.013	0.014	0.028	-0.011	0	-0.008	--	1

Table S2. Correlation loadings of each tract of PCA. For each dMRI measure, PCA on all tracts, association fibres, thalamic radiations and projection fibres were performed respectively. The loadings were reported as correlation loadings.

Tract	FA				MD				ICVF				ISOVF				OD			
	gTotal	gAF	gTR	gPF	gTotal	gAF	gTR	gPF	gTotal	gAF	gTR	gPF	gTotal	gAF	gTR	gPF	gTotal	gAF	gTR	gPF
Parahippocampal part of cingulum	0.361	0.379	--	--	0.472	0.61	--	--	0.604	0.61	--	--	0.63	0.845	--	--	0.791	0.88	--	--
Parahippocampal part of cingulum	0.394	0.419	--	--	0.454	0.587	--	--	0.627	0.636	--	--	0.652	0.865	--	--	0.737	0.846	--	--
Forceps major	0.547	0.552	--	--	0.487	0.518	--	--	0.796	0.793	--	--	0.187	0.122	--	--	0.192	0.127	--	--
Cingulate gyrus part of cingulum	0.558	0.635	--	--	0.61	0.576	--	--	0.783	0.801	--	--	0.346	0.191	--	--	0.216	0.17	--	--
Cingulate gyrus part of cingulum	0.583	0.658	--	--	0.615	0.59	--	--	0.787	0.805	--	--	0.331	0.205	--	--	0.201	0.149	--	--
Uncinate fasciculus	0.649	0.666	--	--	0.665	0.7	--	--	0.808	0.823	--	--	0.422	0.337	--	--	0.483	0.438	--	--
Uncinate fasciculus	0.681	0.696	--	--	0.734	0.75	--	--	0.841	0.856	--	--	0.493	0.356	--	--	0.523	0.481	--	--
Superior longitudinal fasciculus	0.81	0.795	--	--	0.859	0.795	--	--	0.931	0.933	--	--	0.608	0.3	--	--	0.317	0.125	--	--
Superior longitudinal fasciculus	0.824	0.8	--	--	0.847	0.777	--	--	0.928	0.929	--	--	0.622	0.292	--	--	0.356	0.153	--	--
Forceps minor	0.803	0.804	--	--	0.715	0.655	--	--	0.901	0.919	--	--	0.477	0.211	--	--	0.329	0.22	--	--
Inferior longitudinal fasciculus	0.822	0.813	--	--	0.862	0.837	--	--	0.939	0.941	--	--	0.617	0.319	--	--	0.492	0.409	--	--
Inferior longitudinal fasciculus	0.85	0.827	--	--	0.893	0.85	--	--	0.943	0.94	--	--	0.663	0.333	--	--	0.56	0.473	--	--
Inferior fronto-occipital fasciculus	0.827	0.83	--	--	0.871	0.836	--	--	0.946	0.951	--	--	0.624	0.344	--	--	0.461	0.383	--	--
Inferior fronto-occipital fasciculus	0.853	0.842	--	--	0.89	0.849	--	--	0.951	0.955	--	--	0.633	0.332	--	--	0.492	0.398	--	--
Superior thalamic radiation	0.654	--	0.731	--	0.773	--	0.844	--	0.882	--	0.921	--	0.589	--	0.694	--	0.425	--	0.907	--
Superior thalamic radiation	0.65	--	0.737	--	0.742	--	0.82	--	0.874	--	0.916	--	0.562	--	0.674	--	0.445	--	0.915	--
Posterior thalamic radiation	0.675	--	0.785	--	0.756	--	0.863	--	0.872	--	0.902	--	0.626	--	0.879	--	0.436	--	0.271	--
Anterior thalamic radiation	0.774	--	0.807	--	0.82	--	0.85	--	0.902	--	0.933	--	0.573	--	0.585	--	0.495	--	0.558	--
Posterior thalamic radiation	0.693	--	0.812	--	0.774	--	0.88	--	0.879	--	0.906	--	0.656	--	0.897	--	0.456	--	0.33	--
Anterior thalamic radiation	0.773	--	0.816	--	0.811	--	0.847	--	0.895	--	0.931	--	0.572	--	0.597	--	0.503	--	0.655	--
Medial lemniscus	0.262	--	--	0.501	0.253	--	--	-0.328	0.492	--	--	0.737	0.42	--	--	-0.645	0.169	--	--	0.22
Medial lemniscus	0.273	--	--	0.519	0.222	--	--	-0.309	0.48	--	--	0.733	0.379	--	--	-0.63	0.278	--	--	0.258
Middle cerebellar peduncle	0.365	--	--	0.539	0.35	--	--	-0.933	0.503	--	--	0.736	0.355	--	--	-0.753	0.354	--	--	0.862
Acoustic radiation	0.608	--	--	0.577	0.487	--	--	-0.305	0.875	--	--	0.861	0.267	--	--	-0.173	0.255	--	--	0.312
Acoustic radiation	0.618	--	--	0.631	0.505	--	--	-0.274	0.854	--	--	0.854	0.292	--	--	-0.151	0.351	--	--	0.345
Corticospinal tract	0.576	--	--	0.802	0.579	--	--	-0.282	0.789	--	--	0.85	0.319	--	--	-0.152	0.34	--	--	0.552
Corticospinal tract	0.581	--	--	0.821	0.58	--	--	-0.295	0.778	--	--	0.844	0.36	--	--	-0.21	0.402	--	--	0.585

Table S3. Main results for DTI measures (FA and MD). Betas were standardised effect sizes. P values were un-corrected p values. All $p_{corrected}$<0.05 were marked by asterixis. FDR correction was applied on all association tests for a dMRI measure (n = 15*4 measures of depressive symptoms = 60 for tract analysis, and n = 4*4 measures of depressive symptoms = 16 for g analysis). For the measures of depressive symptoms: Cross-sectional measure = depressive level at the imaging assessment, Mean = mean level of depressive symptoms based on multiple assessments for at least two times, Variability = standard deviation of depressive level of multiple assessments for at least three times, and Longitudinal slope = slope of longitudinal changes over all four times of assessments.

		Cross-sectional measure		Mean		Variability		Longitudinal slope	
**Tract name**	**Measure**	**Beta (Std)**	**p**	**Beta (Std)**	**p**	**Beta (Std)**	**p**	**Beta (Std)**	**p**
g.Total	FA	-0.011 (0.007)	0.116	-0.012 (0.007)	0.099	-0.017 (0.008)	0.036	-0.006 (0.014)	0.679
	MD	0.024 (0.007)	4.17e-04*	0.018 (0.007)	0.008*	0.015 (0.007)	0.052	0.04 (0.014)	0.003*
g.AF	FA	-0.009 (0.007)	0.196	-0.01 (0.007)	0.145	-0.016 (0.008)	0.047	-0.012 (0.014)	0.397
	MD	0.019 (0.007)	0.005*	0.014 (0.007)	0.048	0.01 (0.008)	0.192	0.039 (0.014)	0.005*
g.TR	FA	-0.019 (0.007)	0.01	-0.021 (0.007)	0.004*	-0.022 (0.008)	0.006*	-0.001 (0.015)	0.927
	MD	0.022 (0.006)	4.37e-04*	0.018 (0.006)	0.004*	0.015 (0.007)	0.039	0.031 (0.013)	0.017*
g.PF	FA	-0.004 (0.007)	0.611	-9.55e-04 (0.007)	0.895	-0.007 (0.008)	0.371	0.013 (0.014)	0.35
	MD	0.022 (0.007)	0.002*	0.02 (0.007)	0.004*	0.029 (0.008)	2.29e-04*	0.022 (0.014)	0.119
Acoustic radiation	FA	-0.004 (0.006)	0.564	1.60e-04 (0.006)	0.98	-0.002 (0.007)	0.787	-0.003 (0.013)	0.785
	MD	0.015 (0.006)	0.014*	0.008 (0.006)	0.222	5.81e-04 (0.007)	0.933	0.02 (0.013)	0.113
Anterior thalamic radiation	FA	-0.018 (0.007)	0.009	-0.018 (0.007)	0.008	-0.021 (0.008)	0.006	5.61e-04 (0.014)	0.967
	MD	0.028 (0.006)	4.88e-06*	0.029 (0.006)	4.20e-06*	0.02 (0.007)	0.003*	0.03 (0.013)	0.02*
Cingulate gyrus part of cingulum	FA	0.003 (0.006)	0.638	0.001 (0.006)	0.876	-0.006 (0.007)	0.378	-0.014 (0.013)	0.271
	MD	0.017 (0.007)	0.01*	0.008 (0.007)	0.241	0.007 (0.007)	0.294	0.027 (0.013)	0.037
Parahippocampal part of cingulum	FA	-1.10e-04 (0.006)	0.986	-6.36e-04 (0.006)	0.921	-0.01 (0.007)	0.175	0.006 (0.013)	0.621
	MD	0.007 (0.006)	0.256	0.004 (0.006)	0.554	0.004 (0.007)	0.546	0.017 (0.012)	0.17
Corticospinal tract	FA	-0.003 (0.007)	0.699	-7.23e-04 (0.007)	0.915	-0.002 (0.008)	0.838	0.006 (0.014)	0.679
	MD	0.02 (0.007)	0.003*	0.014 (0.007)	0.046	0.012 (0.007)	0.11	0.035 (0.013)	0.007*
Inferior fronto occipital fasciculus	FA	-0.01 (0.007)	0.133	-0.014 (0.007)	0.053	-0.013 (0.008)	0.085	-0.011 (0.014)	0.451
	MD	0.017 (0.007)	0.011*	0.012 (0.007)	0.064	0.006 (0.007)	0.412	0.038 (0.013)	0.004*
Inferior longitudinal fasciculus	FA	-0.018 (0.007)	0.009	-0.02 (0.007)	0.003	-0.015 (0.008)	0.052	0.002 (0.014)	0.911
	MD	0.016 (0.007)	0.013*	0.01 (0.007)	0.117	0.002 (0.007)	0.755	0.033 (0.013)	0.013*
Medial lemniscus	FA	-0.008 (0.006)	0.204	-0.008 (0.006)	0.201	-0.009 (0.007)	0.194	0.003 (0.012)	0.833
	MD	-1.75e-04 (0.006)	0.978	8.64e-04 (0.006)	0.891	0.008 (0.007)	0.229	0.009 (0.012)	0.465
Posterior thalamic radiation	FA	-0.024 (0.007)	3.75e-04*	-0.029 (0.007)	1.31e-05*	-0.022 (0.007)	0.003	0.007 (0.014)	0.62
	MD	0.012 (0.006)	0.049	0.007 (0.006)	0.256	0.006 (0.007)	0.357	0.023 (0.013)	0.075
Superior longitudinal fasciculus	FA	-0.011 (0.007)	0.098	-0.009 (0.007)	0.201	-0.013 (0.008)	0.091	-0.016 (0.014)	0.24
	MD	0.022 (0.007)	0.001*	0.017 (0.007)	0.015*	0.01 (0.008)	0.18	0.031 (0.014)	0.024
Superior thalamic radiation	FA	0.002 (0.007)	0.794	0.004 (0.007)	0.615	-0.007 (0.008)	0.369	-0.014 (0.014)	0.344
	MD	0.02 (0.006)	7.28e-04*	0.016 (0.006)	0.009*	0.015 (0.007)	0.026	0.031 (0.013)	0.013*
Uncinate fasciculus	FA	-8.67e-04 (0.006)	0.893	6.92e-04 (0.006)	0.915	-0.008 (0.007)	0.281	-0.005 (0.013)	0.723
	MD	0.016 (0.006)	0.009*	0.015 (0.006)	0.017*	0.013 (0.007)	0.062	0.032 (0.012)	0.01*
Forceps major	FA	-0.015 (0.007)	0.038	-0.019 (0.007)	0.008	-0.018 (0.008)	0.029	-0.006 (0.015)	0.684
	MD	0.011 (0.007)	0.11	0.014 (0.007)	0.049	0.015 (0.008)	0.06	0.02 (0.014)	0.166
Forceps minor	FA	-0.019 (0.007)	0.009	-0.015 (0.007)	0.037	-0.011 (0.008)	0.181	-0.024 (0.014)	0.102
	MD	0.011 (0.007)	0.114	0.003 (0.007)	0.635	-7.27e-04 (0.008)	0.924	0.037 (0.014)	0.009*
Middle cerebellar peduncle	FA	0.008 (0.007)	0.285	0.009 (0.007)	0.238	-0.007 (0.008)	0.403	0.036 (0.015)	0.014
	MD	0.02 (0.007)	0.006*	0.019 (0.007)	0.008*	0.029 (0.008)	3.87e-04*	0.015 (0.014)	0.301

Table S4. Comparing models with and without the cross-sectional measure. H0 model: Imaging variables~covariates+Mean+Variability, and H1 model: Imaging variables~covariates+Mean+Variability+Cross-sectional measure. ANOVA was utilised to test if adding the cross-sectional measure as an independent variable gives significantly larger variance explained by the model compared to the H0 model. Dependent variables were MD in the tract categories/tracts that were found associated with the cross-sectional measure. Significant p values are highlighted in red.

Method	Model	Independent variable(s)	Dependent variable	RSS	F test Df	F	p
glm	H0	Mean+Variability	g.MD.Total	11707.16	--	--	--
	H1	Mean+Variability+Cross-sectional	g.MD.Total	11700.27	1, 14686	8.659143	0.003259
	H0	Mean+Variability	g.MD.AF	12311.36	--	--	--
	H1	Mean+Variability+Cross-sectional	g.MD.AF	12304.74	1, 14686	7.902749	0.004943
	H0	Mean+Variability	g.MD.TR	10495.86	--	--	--
	H1	Mean+Variability+Cross-sectional	g.MD.TR	10491.75	1, 14686	5.745006	0.016548
	H0	Mean+Variability	g.MD.PF	13164.31	--	--	--
	H1	Mean+Variability+Cross-sectional	g.MD.PF	13162.36	1, 14686	2.180021	0.139834
	H0	Mean+Variability	Middle Cerebellar Peduncle	13402.99	--	--	--
	H1	Mean+Variability+Cross-sectional	Middle Cerebellar Peduncle	13402.25	1, 14686	0.820329	0.365099
				**Deviance**	**Chi test Df**	**Chisq**	**p**
lme	H0	Mean+Variability	Anterior Thalamic Radiation	53866.2	--	--	--
	H1	Mean+Variability+Cross-sectional	Anterior Thalamic Radiation	53864.45	1	1.747914	0.186139
	H0	Mean+Variability	Uncinate Fasciculus	65881.34	--	--	--
	H1	Mean+Variability+Cross-sectional	Uncinate Fasciculus	65878.74	1	2.600238	0.106848
	H0	Mean+Variability	Superior Longitudinal Fasciculus	60468.18	--	--	--
	H1	Mean+Variability+Cross-sectional	Superior Longitudinal Fasciculus	60462.46	1	5.726051	0.016715
	H0	Mean+Variability	Inferior Fronto Occipital Fasciculus	64253.47	--	--	--
	H1	Mean+Variability+Cross-sectional	Inferior Fronto Occipital Fasciculus	64249.67	1	3.798664	0.051293
	H0	Mean+Variability	Superior Thalamic Radiation	56451.53	--	--	--
	H1	Mean+Variability+Cross-sectional	Superior Thalamic Radiation	56444.53	1	6.99626	0.008168
	H0	Mean+Variability	Inferior Longitudinal Fasciculus	61972.76	--	--	--
	H1	Mean+Variability+Cross-sectional	Inferior Longitudinal Fasciculus	61966.72	1	6.037542	0.014005
	H0	Mean+Variability	Corticospinal Tract	71746.14	--	--	--
	H1	Mean+Variability+Cross-sectional	Corticospinal Tract	71737.03	1	9.117795	0.002531
	H0	Mean+Variability	Acoustic Radiation	79033.73	--	--	--
	H1	Mean+Variability+Cross-sectional	Acoustic Radiation	79027.2	1	6.535737	0.010573
	H0	Mean+Variability	Cingulate Gyrus Part Of Cingulum	64031.44	--	--	--
	H1	Mean+Variability+Cross-sectional	Cingulate Gyrus Part Of Cingulum	64022.83	1	8.611179	0.003341

Table S5. Comparing models with and without the longitudinal measures. H0 model: Imaging variables~covariates+Cross-sectional measure, and H1 model: Imaging variables~covariates+Mean+Variability+Cross-sectional measure. ANOVA was utilised to test if adding the cross-sectional measure as an independent variable gives significantly larger variance explained by the model compared to the H0 model. Dependent variables were MD in the tract categories/tracts that were found associated with the cross-sectional measure. Significant p values are highlighted in red.

Method	Model	Independent variable(s)	Dependent variable	RSS	F test Df	F	p
glm	H0	Cross-sectional	g.MD.Total	11701.79	--	--	--
	H1	Mean+Variability+Cross-sectional	g.MD.Total	11700.27	2, 14686	0.959116	0.383256
	H0	Cross-sectional	g.MD.AF	12306.15	--	--	--
	H1	Mean+Variability+Cross-sectional	g.MD.AF	12304.74	2, 14686	0.840598	0.431473
	H0	Cross-sectional	g.MD.TR	10493.18	--	--	--
	H1	Mean+Variability+Cross-sectional	g.MD.TR	10491.75	2, 14686	0.997852	0.368695
	H0	Cross-sectional	g.MD.PF	13172.09	--	--	--
	H1	Mean+Variability+Cross-sectional	g.MD.PF	13162.36	2, 14686	5.430353	0.00439
	H0	Cross-sectional	Middle Cerebellar Peduncle	13410.69	--	--	--
	H1	Mean+Variability+Cross-sectional	Middle Cerebellar Peduncle	13402.25	2, 14686	4.625691	0.009811
lme				**Deviance**	**Chi Df**	**Chisq**	**Pr(>Chisq)**
	H0	Cross-sectional	Anterior Thalamic Radiation	53866.22	--	--	--
	H1	Mean+Variability+Cross-sectional	Anterior Thalamic Radiation	53864.45	2	1.776644	0.411345
	H0	Cross-sectional	Uncinate Fasciculus	65878.81	--	--	--
	H1	Mean+Variability+Cross-sectional	Uncinate Fasciculus	65878.74	2	0.070895	0.965174
	H0	Cross-sectional	Superior Longitudinal Fasciculus	60463.21	--	--	--
	H1	Mean+Variability+Cross-sectional	Superior Longitudinal Fasciculus	60462.46	2	0.757896	0.684581
	H0	Cross-sectional	Inferior Fronto Occipital Fasciculus	64250.01	--	--	--
	H1	Mean+Variability+Cross-sectional	Inferior Fronto Occipital Fasciculus	64249.67	2	0.34637	0.840982
	H0	Cross-sectional	Superior Thalamic Radiation	56447.14	--	--	--
	H1	Mean+Variability+Cross-sectional	Superior Thalamic Radiation	56444.53	2	2.609169	0.271285
	H0	Cross-sectional	Inferior Longitudinal Fasciculus	61968.44	--	--	--
	H1	Mean+Variability+Cross-sectional	Inferior Longitudinal Fasciculus	61966.72	2	1.718461	0.423488
	H0	Cross-sectional	Corticospinal Tract	71740.46	--	--	--
	H1	Mean+Variability+Cross-sectional	Corticospinal Tract	71737.03	2	3.428328	0.180114
	H0	Cross-sectional	Acoustic Radiation	79029.9	--	--	--
	H1	Mean+Variability+Cross-sectional	Acoustic Radiation	79027.2	2	2.705615	0.258514
	H0	Cross-sectional	Cingulate Gyrus Part Of Cingulum	64025.77	--	--	--
	H1	Mean+Variability+Cross-sectional	Cingulate Gyrus Part Of Cingulum	64022.83	2	2.939637	0.229967

Table S6. The effects of stressful life events (SLE), neuroticism, smoking status, and alcohol consumption as covariates for DTI measures. The test was conducted on the full sample of IDP from UK Biobank imaging team, without outlier exclusion or phenotypic data merging. Betas were standardised effect sizes. P values were un-corrected p values. All $p_{corrected}<0.05$ were marked by asterixis. FDR correction was applied on all tests conducted on one dMRI measure.

		Alcohol consumption		Smoking		SLE	
**Tract name**	**Measure**	**Beta (Std)**	**p**	**Beta (Std)**	**p**	**Beta (Std)**	**p**
g.Total	FA	-0.014 (0.007)	0.053	-0.016 (0.007)	0.02*	-0.015 (0.007)	0.031
	MD	0.046 (0.007)	1.04e-11*	0.034 (0.007)	2.31e-07*	0.007 (0.007)	0.316
g.AF	FA	-0.023 (0.007)	0.002*	-0.021 (0.007)	0.003*	-0.014 (0.007)	0.038
	MD	0.034 (0.007)	6.87e-07*	0.022 (0.007)	8.80e-04*	0.006 (0.007)	0.336
g.TR	FA	-0.024 (0.007)	0.001*	-0.031 (0.007)	7.17e-06*	-0.015 (0.007)	0.034
	MD	0.057 (0.006)	7.68e-19*	0.049 (0.006)	1.36e-15*	0.006 (0.006)	0.349
g.PF	FA	0.03 (0.007)	2.13e-05*	0.017 (0.007)	0.012*	-0.009 (0.007)	0.191
	MD	0.05 (0.007)	1.77e-12*	0.052 (0.007)	2.53e-14*	-2.58e-04 (0.007)	0.97
Acoustic radiation	FA	0.009 (0.006)	0.151	0.018 (0.006)	0.003*	-0.01 (0.006)	0.089
	MD	-2.94e-04 (0.006)	0.963	-0.01 (0.006)	0.09	0.005 (0.006)	0.386
Anterior thalamic radiation	FA	-0.022 (0.007)	0.001*	-0.009 (0.007)	0.161	-0.009 (0.007)	0.155
	MD	0.038 (0.006)	1.69e-09*	0.028 (0.006)	2.94e-06*	0.007 (0.006)	0.272
Cingulate gyrus part of cingulum	FA	-0.027 (0.006)	2.23e-05*	-0.024 (0.006)	9.46e-05*	-0.004 (0.006)	0.481
	MD	0.013 (0.007)	0.049	0.011 (0.006)	0.097	0.009 (0.006)	0.15
Parahippocampal part of cingulum	FA	0.006 (0.006)	0.381	6.89e-04 (0.006)	0.911	-0.002 (0.006)	0.691
	MD	0.012 (0.006)	0.068	0.013 (0.006)	0.038	-0.001 (0.006)	0.835
Corticospinal tract	FA	0.027 (0.007)	8.18e-05*	0.01 (0.007)	0.112	-0.004 (0.007)	0.52
	MD	0.021 (0.007)	0.002*	8.87e-04 (0.007)	0.894	0.006 (0.007)	0.392
Inferior fronto occipital fasciculus	FA	-0.009 (0.007)	0.182	-0.013 (0.007)	0.049	-0.009 (0.007)	0.187
	MD	0.036 (0.007)	8.81e-08*	0.017 (0.006)	0.008*	0.005 (0.006)	0.431
Inferior longitudinal fasciculus	FA	-0.019 (0.007)	0.005*	-0.024 (0.007)	2.55e-04*	-0.011 (0.007)	0.109
	MD	0.039 (0.007)	4.39e-09*	0.021 (0.006)	9.27e-04*	0.004 (0.006)	0.547
Medial lemniscus	FA	0.014 (0.006)	0.022*	0.002 (0.006)	0.761	-0.004 (0.006)	0.548
	MD	0.006 (0.006)	0.309	0.001 (0.006)	0.848	-0.011 (0.006)	0.08
Posterior thalamic radiation	FA	-0.03 (0.007)	8.07e-06*	-0.045 (0.006)	4.79e-12*	-0.015 (0.007)	0.023
	MD	0.059 (0.006)	1.65e-20*	0.051 (0.006)	4.74e-17*	0.003 (0.006)	0.607
Superior longitudinal fasciculus	FA	-0.017 (0.007)	0.013*	-0.013 (0.007)	0.047	-0.017 (0.007)	0.014
	MD	0.034 (0.007)	5.70e-07*	0.022 (0.007)	6.86e-04*	0.008 (0.007)	0.25
Superior thalamic radiation	FA	0.001 (0.007)	0.856	-0.012 (0.007)	0.093	-0.009 (0.007)	0.178
	MD	0.038 (0.006)	4.31e-10*	0.039 (0.006)	1.98e-11*	0.007 (0.006)	0.24
Uncinate fasciculus	FA	-0.016 (0.006)	0.014*	-6.78e-05 (0.006)	0.991	-0.015 (0.006)	0.02
	MD	0.026 (0.006)	1.90e-05*	0.01 (0.006)	0.098	0.007 (0.006)	0.257
Forceps major	FA	-0.003 (0.007)	0.719	-0.013 (0.007)	0.06	-0.025 (0.007)	3.44e-04*
	MD	0.017 (0.007)	0.017*	0.011 (0.007)	0.105	0.015 (0.007)	0.031
Forceps minor	FA	-0.027 (0.007)	1.40e-04*	-0.015 (0.007)	0.027	-0.009 (0.007)	0.216
	MD	0.031 (0.007)	5.39e-06*	0.023 (0.007)	5.83e-04*	0.006 (0.007)	0.336
Middle cerebellar peduncle	FA	0.026 (0.007)	5.25e-04*	0.015 (0.007)	0.034	-0.005 (0.007)	0.465
	MD	0.051 (0.007)	1.01e-12*	0.059 (0.007)	2.88e-17*	0.001 (0.007)	0.88

Table S7. The effects of stressful life events (SLE), neuroticism, smoking status, and alcohol consumption as covariates for NODDI measures. The test was conducted on the full sample of IDP from UK Biobank imaging team, without outlier exclusion or phenotypic data merging Betas were standardised effect sizes. P values were un-corrected p values. All $p_{corrected}$<0.05 were marked by asterixis. FDR correction was applied on all the tests for a dMRI measure.

		**Alcohol consumption**		**Smoking**		**SLE**	
**Tract name**	**Measure**	**Beta (Std)**	**p**	**Beta (Std)**	**p**	**Beta (Std)**	**p**
	ICVF	-0.032 (0.007)	6.28e-06*	-0.023 (0.007)	7.70e-04*	-0.009 (0.007)	0.205
g.Total	ISOVF	0.036 (0.007)	8.40e-08*	0.023 (0.007)	3.80e-04*	0.004 (0.007)	0.573
	OD	-0.029 (0.007)	2.65e-05*	-0.009 (0.007)	0.164	0.008 (0.007)	0.228
	ICVF	-0.034 (0.007)	2.40e-06*	-0.022 (0.007)	0.001*	-0.009 (0.007)	0.214
g.AF	ISOVF	0.012 (0.007)	0.092	0.009 (0.007)	0.178	0.006 (0.007)	0.39
	OD	-0.014 (0.007)	0.041*	0.003 (0.006)	0.675	0.009 (0.007)	0.176
	ICVF	-0.037 (0.007)	1.74e-07*	-0.031 (0.007)	4.50e-06*	-0.009 (0.007)	0.212
g.TR	ISOVF	0.052 (0.007)	9.28e-16*	0.034 (0.006)	5.44e-08*	0.003 (0.006)	0.641
	OD	-0.03 (0.007)	6.89e-06*	-0.013 (0.007)	0.052	0.001 (0.007)	0.855
	ICVF	-0.01 (0.007)	0.171	-0.007 (0.007)	0.283	-0.007 (0.007)	0.296
g.PF	ISOVF	0.043 (0.007)	1.54e-09*	0.044 (0.007)	2.30e-10*	-0.004 (0.007)	0.554
	OD	-0.049 (0.007)	3.97e-12*	-0.041 (0.007)	3.40e-09*	-0.001 (0.007)	0.875
	ICVF	-0.024 (0.007)	6.10e-04*	-0.012 (0.007)	0.08	-0.009 (0.007)	0.162
Acoustic radiation	ISOVF	-0.012 (0.006)	0.046	-0.022 (0.006)	1.93e-04*	0.002 (0.006)	0.715
	OD	-0.02 (0.006)	0.001*	-0.029 (0.006)	8.22e-07*	-0.002 (0.006)	0.739
	ICVF	-0.028 (0.007)	2.92e-05*	-0.011 (0.007)	0.09	-0.007 (0.007)	0.256
Anterior thalamic radiation	ISOVF	0.029 (0.006)	6.19e-06*	0.029 (0.006)	2.87e-06*	0.005 (0.006)	0.393
	OD	3.79e-04 (0.007)	0.955	0.002 (0.007)	0.778	0.002 (0.007)	0.743
	ICVF	-0.027 (0.007)	9.78e-05*	-0.02 (0.007)	0.002*	-0.009 (0.007)	0.197
Cingulate gyrus part of cingulum	ISOVF	-0.008 (0.006)	0.206	2.94e-06 (0.006)	1	0.002 (0.006)	0.701
	OD	0.014 (0.006)	0.021*	0.015 (0.006)	0.011*	2.07e-04 (0.006)	0.972
	ICVF	-0.007 (0.007)	0.318	9.61e-04 (0.006)	0.882	0.004 (0.006)	0.567
Parahippocampal part of cingulum	ISOVF	0.007 (0.006)	0.238	0.008 (0.006)	0.17	0.004 (0.006)	0.504
	OD	-0.011 (0.006)	0.072	0.004 (0.006)	0.524	0.006 (0.006)	0.283
	ICVF	-0.013 (0.007)	0.081	-0.017 (0.007)	0.015*	-0.01 (0.007)	0.151
Corticospinal tract	ISOVF	0.011 (0.007)	0.113	-0.006 (0.006)	0.373	0.001 (0.006)	0.832
	OD	-0.044 (0.007)	3.52e-11*	-0.026 (0.006)	5.85e-05*	-0.005 (0.006)	0.421
Inferior fronto occipital fasciculus	ICVF	-0.031 (0.007)	8.86e-06*	-0.022 (0.007)	0.001*	-0.008 (0.007)	0.264
	ISOVF	0.019 (0.007)	0.006*	-0.003 (0.007)	0.704	0.001 (0.007)	0.848

		Alcohol consumption		Smoking		SLE	
**Tract name**	**Measure**	**Beta (Std)**	**p**	**Beta (Std)**	**p**	**Beta (Std)**	**p**
	OD	-0.029 (0.007)	1.55e-05*	-0.015 (0.006)	0.019*	0.002 (0.006)	0.811
	ICVF	-0.03 (0.007)	2.28e-05*	-0.024 (0.007)	3.95e-04*	-0.007 (0.007)	0.329
Inferior longitudinal fasciculus	ISOVF	0.029 (0.007)	2.17e-05*	-0.001 (0.007)	0.867	-2.40e-05 (0.007)	0.997
	OD	-0.018 (0.007)	0.005*	0.003 (0.006)	0.582	0.005 (0.006)	0.442
	ICVF	0.021 (0.006)	5.27e-04*	0.01 (0.006)	0.104	0.004 (0.006)	0.452
Medial lemniscus	ISOVF	0.013 (0.006)	0.046	0.004 (0.006)	0.526	-0.008 (0.006)	0.163
	OD	0.009 (0.007)	0.207	0.019 (0.007)	0.003*	0.008 (0.007)	0.239
	ICVF	-0.035 (0.007)	6.42e-07*	-0.038 (0.007)	1.34e-08*	-0.007 (0.007)	0.315
Posterior thalamic radiation	ISOVF	0.051 (0.006)	1.41e-15*	0.03 (0.006)	1.50e-06*	0.001 (0.006)	0.838
	OD	-0.013 (0.006)	0.029*	0.009 (0.006)	0.115	0.01 (0.006)	0.097
	ICVF	-0.034 (0.007)	2.93e-06*	-0.022 (0.007)	0.001*	-0.009 (0.007)	0.174
Superior longitudinal fasciculus	ISOVF	0.018 (0.007)	0.008*	0.007 (0.006)	0.269	0.004 (0.006)	0.546
	OD	-0.027 (0.006)	1.27e-05*	-0.013 (0.006)	0.027*	0.009 (0.006)	0.143
	ICVF	-0.039 (0.007)	2.27e-08*	-0.038 (0.007)	1.80e-08*	-0.009 (0.007)	0.162
Superior thalamic radiation	ISOVF	0.02 (0.006)	5.87e-04*	0.017 (0.006)	0.002*	0.004 (0.006)	0.455
	OD	-0.034 (0.007)	4.04e-07*	-0.016 (0.006)	0.01*	-7.12e-04 (0.006)	0.912
	ICVF	-0.033 (0.007)	1.17e-06*	-0.016 (0.006)	0.014*	-0.01 (0.006)	0.13
Uncinate fasciculus	ISOVF	0.006 (0.007)	0.391	-0.003 (0.006)	0.579	0.007 (0.006)	0.294
	OD	-0.002 (0.006)	0.798	-0.002 (0.006)	0.796	0.011 (0.006)	0.068
	ICVF	-0.03 (0.007)	4.18e-05*	-0.024 (0.007)	6.38e-04*	-0.014 (0.007)	0.052
Forceps major	ISOVF	0.003 (0.007)	0.724	-0.003 (0.007)	0.704	0.013 (0.007)	0.055
	OD	-0.034 (0.007)	2.49e-06*	-0.019 (0.007)	0.006*	0.017 (0.007)	0.014
	ICVF	-0.035 (0.007)	8.30e-07*	-0.017 (0.007)	0.015*	-0.005 (0.007)	0.495
Forceps minor	ISOVF	0.003 (0.007)	0.676	0.014 (0.007)	0.042	0.008 (0.007)	0.254
	OD	-0.014 (0.007)	0.052	-0.002 (0.007)	0.751	-6.06e-04 (0.007)	0.932
	ICVF	-6.47e-04 (0.007)	0.926	0.004 (0.007)	0.553	-0.004 (0.007)	0.603
Middle cerebellar peduncle	ISOVF	0.047 (0.007)	1.42e-10*	0.057 (0.007)	4.16e-16*	0.002 (0.007)	0.826
	OD	-0.037 (0.007)	3.22e-07*	-0.033 (0.007)	2.30e-06*	4.28e-04 (0.007)	0.951

Table S8. Main results for NODDI measures (ICVF, ISOVF, and OD). Betas were standardised effect sizes. For the abbreviations of measures for depressive symptoms, see the legend of Table S3.

		Cross-sectional measure		Mean		Variability		Longitudinal measure	
**Tract name**	**Measure**	**Beta (Std)**	**p**	**Beta (Std)**	**p**	**Beta (Std)**	**p**	**Beta (Std)**	**p**
	ICVF	-0.007 (0.007)	0.313	-0.003 (0.007)	0.636	-0.003 (0.008)	0.696	-0.024 (0.014)	0.094
g.Total	ISOVF	0.029 (0.007)	1.49e-05*	0.027 (0.007)	6.16e-05*	0.027 (0.007)	2.54e-04*	0.025 (0.014)	0.062
	OD	-0.001 (0.007)	0.86	0.008 (0.007)	0.233	0.019 (0.008)	0.011*	-0.035 (0.014)	0.011*
	ICVF	-0.007 (0.007)	0.337	-0.003 (0.007)	0.669	-0.004 (0.008)	0.65	-0.028 (0.014)	0.057
g.AF	ISOVF	0.022 (0.007)	0.002*	0.022 (0.007)	0.003*	0.02 (0.008)	0.012*	0.01 (0.014)	0.495
	OD	0.007 (0.007)	0.285	0.016 (0.007)	0.018*	0.023 (0.008)	0.003*	-0.026 (0.014)	0.056
	ICVF	-0.012 (0.007)	0.098	-0.01 (0.007)	0.151	-0.007 (0.008)	0.386	-0.019 (0.014)	0.191
g.TR	ISOVF	0.018 (0.006)	0.007*	0.014 (0.007)	0.034*	0.016 (0.007)	0.029*	0.027 (0.013)	0.042
	OD	-0.016 (0.007)	0.015*	-0.016 (0.007)	0.022*	-4.87e-04 (0.007)	0.948	-0.013 (0.014)	0.326
	ICVF	0.002 (0.007)	0.782	0.009 (0.007)	0.218	0.007 (0.008)	0.401	-0.014 (0.014)	0.328
g.PF	ISOVF	0.02 (0.007)	0.004*	0.022 (0.007)	0.002*	0.033 (0.008)	2.29e-05*	0.014 (0.014)	0.304
	OD	-0.014 (0.007)	0.054	-0.009 (0.007)	0.216	-0.001 (0.008)	0.899	-0.039 (0.014)	0.006*
	ICVF	-0.007 (0.007)	0.315	-0.002 (0.007)	0.797	-7.31e-04 (0.008)	0.925	-0.023 (0.014)	0.11
Acoustic radiation	ISOVF	0.013 (0.006)	0.041	0.009 (0.006)	0.173	0.002 (0.007)	0.739	0.004 (0.013)	0.727
	OD	-0.008 (0.006)	0.185	-0.004 (0.006)	0.461	0.004 (0.007)	0.582	-0.02 (0.012)	0.095
	ICVF	-0.007 (0.007)	0.324	-0.005 (0.007)	0.445	-0.002 (0.008)	0.789	-0.023 (0.014)	0.101
Anterior thalamic radiation	ISOVF	0.039 (0.006)	2.15e-09*	0.042 (0.007)	8.86e-11*	0.037 (0.007)	2.82e-07*	0.015 (0.013)	0.232
	OD	-0.004 (0.007)	0.558	-0.002 (0.007)	0.819	0.014 (0.008)	0.066	-0.031 (0.014)	0.024
	ICVF	0.003 (0.007)	0.691	0.007 (0.007)	0.343	-0.003 (0.008)	0.711	-0.021 (0.014)	0.134
Cingulate gyrus part of cingulum	ISOVF	0.025 (0.006)	6.68e-05*	0.021 (0.006)	0.001*	0.01 (0.007)	0.162	0.009 (0.013)	0.464
	OD	-0.007 (0.006)	0.276	-8.22e-04 (0.006)	0.894	0.003 (0.007)	0.662	0.013 (0.012)	0.304
	ICVF	0.009 (0.007)	0.156	0.016 (0.007)	0.017	0.006 (0.007)	0.424	-0.024 (0.014)	0.074
Parahippocampal part of cingulum	ISOVF	0.015 (0.006)	0.018*	0.014 (0.006)	0.023	0.014 (0.007)	0.039	0.004 (0.012)	0.759
	OD	0.01 (0.006)	0.098	0.016 (0.006)	0.006*	0.02 (0.007)	0.002*	-0.018 (0.012)	0.146
	ICVF	-0.001 (0.007)	0.851	0.006 (0.007)	0.43	0.005 (0.008)	0.523	-0.005 (0.014)	0.725
Corticospinal tract	ISOVF	0.022 (0.007)	0.001*	0.019 (0.007)	0.005*	0.014 (0.007)	0.056	0.036 (0.013)	0.008*
	OD	-0.012 (0.007)	0.06	-0.01 (0.007)	0.117	-0.006 (0.007)	0.415	-0.012 (0.013)	0.343
	ICVF	-0.006 (0.007)	0.358	-0.004 (0.007)	0.554	-0.002 (0.008)	0.773	-0.027 (0.014)	0.059
Inferior fronto occipital fasciculus	ISOVF	0.018 (0.007)	0.009*	0.015 (0.007)	0.032	0.011 (0.008)	0.155	0.022 (0.014)	0.102
	OD	-0.012 (0.007)	0.06	-0.003 (0.007)	0.671	0.007 (0.007)	0.336	-0.025 (0.013)	0.058
	ICVF	-0.009 (0.007)	0.202	-0.006 (0.007)	0.395	-0.003 (0.008)	0.701	-0.027 (0.014)	0.063
Inferior longitudinal fasciculus	ISOVF	0.017 (0.007)	0.013*	0.013 (0.007)	0.052	0.005 (0.008)	0.482	0.015 (0.014)	0.274
	OD	-5.82e-04 (0.006)	0.928	0.009 (0.007)	0.178	0.012 (0.007)	0.082	-0.051 (0.013)	1.35e-04*
	ICVF	0.008 (0.006)	0.18	0.016 (0.006)	0.007	0.013 (0.007)	0.049	-0.006 (0.012)	0.609
Medial lemniscus	ISOVF	0.002 (0.006)	0.765	0.005 (0.006)	0.426	0.011 (0.007)	0.109	0.007 (0.012)	0.551
	OD	0.018 (0.007)	0.006*	0.03 (0.007)	1.14e-05*	0.021 (0.007)	0.006*	-0.023 (0.013)	0.087
	ICVF	-0.012 (0.007)	0.079	-0.012 (0.007)	0.077	-0.007 (0.008)	0.334	-0.01 (0.014)	0.466
Posterior thalamic radiation	ISOVF	0.007 (0.006)	0.285	0.002 (0.006)	0.77	0.006 (0.007)	0.376	0.021 (0.013)	0.106
	OD	0.004 (0.006)	0.554	0.012 (0.006)	0.053	0.013 (0.007)	0.053	-0.033 (0.012)	0.008
	ICVF	-0.015 (0.007)	0.035	-0.011 (0.007)	0.139	-0.008 (0.008)	0.339	-0.023 (0.014)	0.118
Superior longitudinal fasciculus	ISOVF	0.02 (0.007)	0.003*	0.018 (0.007)	0.006*	0.011 (0.007)	0.15	0.024 (0.014)	0.075
	OD	-0.011 (0.006)	0.07	-0.01 (0.006)	0.133	0.005 (0.007)	0.501	-0.007 (0.013)	0.555
	ICVF	-0.013 (0.007)	0.052	-0.01 (0.007)	0.137	-0.01 (0.008)	0.202	-0.019 (0.014)	0.178
Superior thalamic radiation	ISOVF	0.019 (0.006)	0.001*	0.017 (0.006)	0.003*	0.015 (0.006)	0.018*	0.029 (0.012)	0.014*
	OD	-0.018 (0.007)	0.007*	-0.019 (0.007)	0.005*	-0.007 (0.007)	0.338	-1.92e-04 (0.013)	0.988
	ICVF	-0.004 (0.007)	0.535	-0.002 (0.007)	0.817	-0.006 (0.007)	0.402	-0.027 (0.013)	0.047
Uncinate fasciculus	ISOVF	0.027 (0.006)	4.37e-05*	0.029 (0.007)	1.05e-05*	0.024 (0.007)	7.46e-04*	0.01 (0.013)	0.436
	OD	-0.007 (0.006)	0.259	-0.004 (0.006)	0.494	0.004 (0.007)	0.533	-0.027 (0.012)	0.028
	ICVF	-0.014 (0.007)	0.059	-0.014 (0.007)	0.056	-0.008 (0.008)	0.332	-0.023 (0.015)	0.117
Forceps major	ISOVF	0.008 (0.007)	0.293	0.012 (0.007)	0.094	0.015 (0.008)	0.055	0.01 (0.014)	0.495
	OD	-0.007 (0.007)	0.325	-0.002 (0.007)	0.817	0.006 (0.008)	0.469	-0.016 (0.014)	0.262
	ICVF	-0.005 (0.007)	0.465	-0.001 (0.007)	0.871	0.003 (0.008)	0.693	-0.022 (0.014)	0.122
Forceps minor	ISOVF	0.013 (0.007)	0.059	0.009 (0.007)	0.184	0.011 (0.008)	0.169	0.022 (0.014)	0.109
	OD	0.004 (0.007)	0.591	0.003 (0.007)	0.685	0.008 (0.008)	0.343	-0.002 (0.015)	0.91
	ICVF	0.015 (0.007)	0.037	0.016 (0.007)	0.025	0.009 (0.008)	0.254	-0.004 (0.013)	0.781
Middle cerebellar peduncle	ISOVF	0.023 (0.007)	0.002*	0.023 (0.007)	0.002*	0.034 (0.008)	3.58e-05*	0.01 (0.014)	0.485
	OD	-0.012 (0.007)	0.098	-0.01 (0.007)	0.168	-0.003 (0.008)	0.669	-0.033 (0.015)	0.022

Supplemental References

1. Andersson JLR, Jenkinson M, Smith SM (2007): Non-linear optimisation. FMRIB technical report TR07JA1. *In Pract*. .
2. Andersson JLR, Sotiropoulos SN (2015): An integrated approach to correction for off-resonance effects and subject movement in diffusion MR imaging. *Neuroimage*. 125: 1063–1078.
3. Rosseel Y (2012): lavaan : an R package for structural equation modeling and more Version 0 . 5-12 (BETA). *J Stat Softw*. 48: 1–36.
4. Clarke T-K, Adams MJ, Davies G, Howard DM, Hall LS, Padmanabhan S, *et al.* (2017): Genome-wide association study of alcohol consumption and genetic overlap with other health-related traits in UK Biobank (N=112 117). *Mol Psychiatry*. 22: 1376–1384.
5. Smith SM, Nichols TE (2018): Statistical Challenges in “Big Data” Human Neuroimaging NeuroView. *Neuron*. 97: 263–268.
